# Carer and patient experiences in a virtual hospital: service insights from a mixed-methods analysis of reported experience measures

**DOI:** 10.1186/s41687-026-01029-w

**Published:** 2026-03-04

**Authors:** Kanesha Ward, Tim Jackson, Shannon Saad, Jenna Bartyn, Sue Amanatidis, Sarah White, Shila Poudel, Owen Hutchings, Annie Y. S. Lau

**Affiliations:** 1 Centre for Health Informatics, Australian Institute of Health Innovation, Macquarie University, Sydney, NSW 2109 Australia; 2 Sydney Virtual (previously known as rpavirtual), Sydney Local Health District, Sydney, Australia; 3 School of Clinical Medicine - Discipline of General Practice, University of New South Wales, Sydney, Australia; 4 Faculty of Medicine and Health Sciences, Macquarie University, Sydney, Australia

**Keywords:** Carers, Patient Experience, Virtual consultations, Survey, Telehealth, Virtual hospital, Mixed methods

## Abstract

**Background:**

Carers, including family members and companions, play a crucial role in supporting patients and sharing insights about their care experiences. Their influence is especially important in virtual hospital models, where patients receive remote hospital-level care using digital technologies. However, there is limited evidence examining what carers think about virtual hospitals or how their experiences could be improved.

**Methodology:**

We explored Patient-Reported Experience Measure (PREM) surveys completed by 3047 patients and 235 carers to better understand their experiences with virtual hospitals and suggest insights into how to enhance carer and patient experience from the carer’s perspective. Participants were patients and carers discharged from an acute respiratory model of care within a virtual hospital (from 1st March 2020 to 26th August 2024). We conducted both statistical and qualitative (content) analysis. Mann-Whitney U test was used to compare differences between carer and patient responses to 12 Likert scale questions on their reported experience. Content analysis was applied to two free-text comments (What was the best part of the care you received from the virtual hospital? What part of your care provided by virtual hospital most needs improving?). Keywords from the content analysis of free-text responses were integrated with statistical findings from Likert-scale questions to develop insights.

**Results:**

3047 patients and 235 carers of PREM surveys were analysed. The overall rating of the virtual hospital service was the same between carer and patients, with the mean response of ‘Excellent’. Our service insights from carers highlight how they support patients, shape their care experience, and offer valuable feedback. We identified six key insights that improve patient and carer experience including reassurance, effective communication, consistent availability, supportive healthcare workers, person-centred clinical service, and usable technology.

**Conclusions:**

Findings from this study can highlight ways virtual hospital models can be more inclusive and centred around both patients and carers, using data that is already being collected through routine surveys.

**Plain English abstract:**

Carers such as family members and companions play a vital, but often under-recognised role in care by supporting patients emotionally, practically, and through communication. Their influence is especially important in virtual hospital models, where patients receive remote hospital-level care using digital technologies. However, there is limited evidence examining what carers think about virtual hospitals or how their experiences could be improved. In this study, we have explored patient-reported experience measure surveys completed by 3047 patients and 235 carers to better understand their experiences with a virtual hospital service. This study identified service insights from carers that highlight how they support patients, shape their care experience, and offer valuable feedback. We identified six key insights that improve patient and carer experience including reassurance, effective communication, consistent availability, supportive healthcare workers, person-centred clinical service, and usable technology. Findings from this study can help improve virtual hospital models to be more inclusive and centred around both patients and carers, using data that is already being collected as part of standard care.

**Supplementary Information:**

The online version contains supplementary material available at 10.1186/s41687-026-01029-w.

## Introduction

Carers play a vital role in the patient healthcare journey, supporting patients both in clinical settings and at home [1, 2]. In this study, “carers” refers to individuals involved in care (e.g., parents, other family members, unpaid caregivers, partners, friends, paid carers, or other companions) that are not part of the healthcare provider team. Carers can support better communication with healthcare providers to bridge the gap between what the patient experiences and what the healthcare provider needs to know to offer the best care (e.g., patient preferences, limitations, responses to treatment, and patient values) [1, 3]. They also provide a wide range of day-to-day support, including emotional [4] and physical support (e.g., cooking, medications, and showering) [5].

Virtual hospitals deliver hospital-level care at home, supporting both patients and carers as a bridge between in-person hospital care and at home recovery [6, 7]. Virtual hospitals enhance patient care by using digital technology to reduce non-urgent admissions [8], support continuity [9], and comfort within the home environment [9]. Despite carers’ key role and early research into carer burden, limited research has explored carers’ experiences and perspectives of virtual hospitals. While carers often report satisfaction, they also take on additional tasks such as meal preparation, technical support, and assisting with examinations [10]. Carers may be either co-located with or remote from the patient, introducing setting-specific requirements (e.g., an out-of-state or a live-in partner). A comprehensive understanding of the burdens and benefits associated with virtual caregiving is essential for developing targeted interventions (e.g., support mechanisms, carer programs, and operational policies) to mitigate caregiver burden and enhance patient experience [10].

Patient Reported Experience Measure (PREM) surveys are routinely used in healthcare to gather feedback from patients and their carers about their experiences. These post-discharge surveys help assess care quality, highlight problem areas, and support service evaluation and improvement [11, 12]. Carers can also answer PREM surveys and provide feedback on behalf of patients or as an additional perspective. To our knowledge, there is limited research that has used PREM surveys to explore virtual hospital experiences or carer-reported experiences with virtual models of care. The Beryl Institute defines Patient Experience as “the sum of all interactions, shaped by an organisation’s culture, that influence patient perceptions across the continuum of care” [13]. Improving patient experience requires meaningful engagement, shared decision-making, and open communication between all stakeholders [1, 14, 15]. This study demonstrates the feasibility and value of routinely using PREM surveys to support ongoing quality improvement and patient-centred care in virtual settings.

## Methods

### Study aims

This study aims to explore patient and carer-reported experiences from a virtual hospital model of care, identify statistically significant differences between their responses to uncover what matters most to carers, and offer insights into how virtual hospital models can be improved from the carer’s perspective.

### Study population and setting

Participants were from a metropolitan virtual hospital, Sydney Virtual (previously known as **rpa**virtual), in New South Wales, Australia. Sydney Virtual provides hospital-level virtual care for patients who are borderline for in-person ward admission, prefer home care, or require closer monitoring during recovery. Established in 2020 during the onset of the COVID-19 pandemic, Sydney Virtual is the first virtual hospital in Australia. This study is based on the largest PREM surveys dataset collected to date by a virtual hospital.

Participants of this study included patients and their carers involved in the Acute Respiratory model of care referred either through Emergency Departments or their General Practitioners (GPs). The service was rapidly expanded during the COVID-19 lockdowns to manage COVID positive patients (e.g., patients in Medihotels) and further expanded into an Acute Respiratory model of care in October 2022. The Acute Respiratory model offers nursing, medical, and allied health care for patients with mild to moderate respiratory illness (e.g., community-acquired pneumonia, COVID-19, influenza or other viral illness, mild-moderate infective exacerbation of Chronic Obstructive Pulmonary Disease, or Asthma) who are at risk of requiring hospital admission due to respiratory disease severity, compromised health status or intercurrent health conditions.

Over the course of four years (2020–2024), the model of care at Sydney Virtual has evolved significantly, driven in part by feedback from PREM survey data. Many of the concerns raised in earlier phases of Sydney Virtual, particularly during the peak of the COVID-19 period when the model saw its largest patient cohort, were addressed through ongoing service improvements. This model of care was selected because it is well-established and supported by rich patient and carer experience data, making it ideal for exploring carer perspectives in depth.

Data collection methods are detailed in a previously published protocol [16]. Upon admission, patients receive medications and a wearables pack (including an oximeter, thermometer, and patient information). Care includes 24-hour virtual nursing support, daily virtual reviews by phone or video call, email messaging, and other care as required. Most recently, in July 2024 the model of care introduced a remote patient monitoring application, the Miya Care app. The app is prescribed to patients to automatically track wearable device measurements via Bluetooth. It is important to note, trends modelled in this study likely reflect service maturation rather than app introduction given limited exposure (July–Aug 2024).

### Datasets and screening procedures

Two datasets were analysed in this study, namely PREM surveys and Electronic Medical Record (EMR) patient admissions data. The PREM survey dataset captures the reported experiences of patients and carers who completed a survey post-discharge related to the Acute Respiratory model of care within the virtual hospital. The EMR patient admissions data, which is independent of the PREM surveys, provides a summary of demographic and admission details for all patients enrolled in this model of care. Due to anonymity of PREM responses, linkage to EMR-level demographics was not possible. Therefore, respondent versus non-respondent characteristics could not be compared. The influence of demographic or health characteristics on responses could also not be assessed (i.e., no case-mix adjustment or stratification by demographic variables was possible). The EMR patient admissions dataset offers a broader demographic context for interpreting the experiences reported in the PREM surveys.

#### EMR patient admissions

A de-identified EMR census dataset of the virtual hospital Acute Respiratory model of care was extracted containing demographic details of 20,489 patients. Variables include the patient’s age, gender, length of admission, preferred language, Aboriginal and/or Torres Strait Islander status, and country of birth. We filtered the data to limit patient admissions to between the 1 March 2020 and 26 August 2024 to align with the collected PREM survey period.

#### Patient-reported experience measure (PREM) survey

The PREM survey was distributed via the REDCap data collection application to patients of Sydney Virtual Acute Respiratory model of care a week after discharge. It included 36 questions: five on demographic details and 31 focused on reported experiences based on the eight principles of person-centred care by the PICKER institute Europe [17]. The eight principles include communication, continuity of care, involvement in decision and respect, attention to physical and environmental needs, emotional support, support for family and carers, effective treatment, and fast access to reliable healthcare advice. Survey domains included technology use, access to care, overall satisfaction, information and education, trust and confidence, co-ordination of care, and willingness to recommend the service. Responses were a mix of Yes/No, multiple choice, 4- or 5-point Likert scale items, and free text questions. The survey was developed and implemented by Sydney Virtual, and routinely collected as part of organisational practices.

We analysed a dataset of PREM surveys collected from the model of care between the 1 March 2020 and 26 August 2024. In total, 3352 responses were screened to differentiate between patients and carers. Carers were identified via a demographic question to indicate the person completing the survey (parent, carer, or cultural support worker). Of 3352 responses, we analysed 235 carer surveys and 3047 patient surveys. KW conducted a screening of all responses and then independently verified by AL and TJ.

### Data analysis

#### Statistical analysis

Likert items were summarised using median and interquartile range [IQR]. Mann-Whitney U test was used to compare differences in PREM surveys between carers and patients using IBM Statistical Package for the Social Sciences (SPSS; Version 30, IBM Corp). Statistical significance was set at *P* < 0.05 for all analyses, with 95% confidence intervals reported. For the Mann-Whitney U test, the null hypothesis is the distribution of question responses is the same across carers and patients. Ordinal effect sizes (Cliff’s delta) with 95% confidence intervals were calculated for each Likert item. A single primary endpoint was pre-specified (Question item 1: Overall, how would you rate the care you received from Sydney Virtual?). The remaining 11 Likert items were considered secondary. To control for multiple comparisons and false positives, p-values for secondary items were adjusted using the Benjamini–Hochberg false discovery rate (FDR) procedure (α = 0.05). Adjusted p-values are reported, and statistically significant findings after FDR correction are denoted in Table 2. To account for substantial changes to the model of care, PREM response averages were presented in Supplementary Table 1 per year and visually presented in Figs. 2 and 3.

Missing data were handled using a per-item complete-case approach. Participants were retained in the dataset, and analyses for each Likert item were conducted using all available responses for that item. No imputation or category collapsing was performed. Denominators (n) for each item are reported in Table 2.

Descriptive analysis was conducted to obtain insights into the EMR demographics dataset, as well as how patients’ and carers’ responses to each PREM survey question changes over time. Microsoft Excel was used to clean and manage both datasets.

#### Content analysis of free-text responses of carers

Content analysis was conducted on carer responses from the two free-text PREM survey questions: What was the best part of the care you received from Sydney Virtual? and What part of your care provided Sydney Virtual most needs improving? The content analysis was underpinned by a constructivist qualitative framework, acknowledging that carers’ perspectives are shaped by their lived experiences and interpreted through researcher analysis.

An inductive, data-driven coding approach was used to identify patterns and group similar reported experiences. Common keywords and recommendation statements served as a starting point to interrogate the data representing areas important to carers. Common function words (e.g., “the”, “and”, “is”) were excluded, while adjectives (e.g., “excellent”) were retained for their descriptive value. For frequently used terms, we retained the original wording in the cleaned keyword lists to reflect carers’ specific language and meaning. However, for lower-frequency terms (e.g., “daily contact” and “daily communication”) that conveyed similar meanings, we grouped them under broader conceptual categories during data cleaning as a pragmatic approach to streamline the visualisation in the word cloud while preserving key distinctions in the data.

Two researchers (KW and TJ) coded the data using a consensus process rather than independent parallel coding. Codes were discussed in iterative meetings and discussion until full consensus was reached, thus measures of inter-coder reliability (e.g., Cohen’s κ) were not applicable. Disagreements were addressed with a third senior researcher (AL). The coding framework was developed (see Supplementary Tables 2 and 3 for supporting quotes).

Cleaned keyword lists were transferred into separate Word documents for each question. Word clouds were generated using wordclouds.com to visually represent frequently used words and phrases of the cleaned keyword lists [18]. Files were managed using NVivo and Microsoft Excel. Due to the brevity of responses (Minimum = 1 word; Maximum = 270 words; AVG = 13 words), this process was classified as content analysis rather than thematic analysis.

#### Insights into improving patient experience from carers’ perspective

Common keywords and recommendation statements identified through word clouds were used as a starting point to explore the data and uncover insights into areas of importance to carers. Keywords from the content analysis of free-text responses were integrated with statistical findings from Likert-scale questions to develop overarching themes, which served as insight categories. We did analyse both positive and negative feedback about the same aspects of care (e.g., access, communication). Contrasting views were noted and compared during coding and interpretation of data. These insights informed our recommendations and conclusions on how to improve both carer and patient experiences, specifically from the carer’s perspective. The results were presented in a table format.

## Results

### Demographics

There were 20,390 patient admissions between the period of 1 March 2020 and 26 August 2024. 3352 PREM surveys were completed during this period, indicating a response rate of 16.44%.

The average age of patients admitted to the virtual hospital was 37 years, and the average length of admission was 12 days. 45.21% (9263/20,390) patients were male, 54.15% (11,094/20,390) were female, and 0.16% (33/20,390) were undisclosed genders. 5.28% (1082/20,390) identified as Aboriginal and/or Torres Strait Islander. 20.17% (4112/20,390) of patients spoke a language other than English. Table 1 provides a breakdown of the ages of the patients.

Although the PREM survey included optional demographic questions, completion rates for these items were extremely low (e.g., 4% of 3,352 PREM responses provided age). PREM surveys were completed anonymously. To maintain data integrity, only EMR-level demographics are reported, and PREM demographic comparisons were not feasible. Aggregate demographic data for the entire admissions cohort are presented in Table 1 to provide contextual information.

**Table 1:** Age demographics of admitted patients (*n* = 20390)

Age group	*n* (%)
Under 5 years	777 (3.79)
5–9 years	721 (3.52)
10–17 years	1275 (6.22)
18–29 years	3740 (18.31)
30–49 years	5974 (29.30)
50–64 years	3044 (14.93)
65–79 years	3347 (16.41)
80 + years	1518 (7.45)

### PREM survey results

After PREM surveys were screened, we included 235 carer surveys and 3047 patient surveys for analysis. The results demonstrate PREM survey responses from participants were highly positive. Responses to each Likert scale question are provided in Table 2 with the median response (MED), interquartile range (IQR), p-values calculated from the Mann-Whitney U test, false discovery rate using the Benjamini-Hochberg procedure (FDR), and Cliff’s delta effect sizes with 95% CIs to calculate the distribution of responses between patients and carers. After applying the Benjamini–Hochberg FDR correction to adjust for multiple comparisons, 7 of the 12 Likert items remained statistically significant (adjusted *p* ≤ 0.014).

**Table 2:** Characteristics of PREM survey responses of Carers (*n* = 235) and Patients (*n* = 3047)

#	Question	Score	Text	%	Carer		Patient		*P* value^1^	Adjusted *p* value *(FDR)*	Cliff’s δ (95% CI)
					MED	IQR	MED	IQR			
1	Overall, how would you rate the care you received from Sydney Virtual? (Patient *n* = 3030; Carer *n* = 232)										
		4	Excellent	72.13	4	0 [4–4]	4	1 [3–4]	0.182	0.243	-0.041 (-0.095-0.016)
		3	Good	18.42							
		2	Fair	4.51							
		1	Poor	2.12							
		0	Very Poor	2.82							
2	My (or the person I care for) healthcare needs were met) (Patient *n* = 2997; Carer *n* = 288)										
		4	Always	79.84	4	0 [4–4]	4	0 [4–4]	**< 0.001**	**0.004**	-0.076 (-0.122–0.028)
		3	Mostly	0.78							
		2	Sometimes	13.71							
		1	Rarely	0.03							
		0	Never	5.64							
3	The care and treatment I received from Sydney Virtual helped me (or the person I care for) (Patient *n* = 3019; Carer *n* = 233)										
		4	Strongly agree	70.85	4	0 [4–4]	4	1 [3–4]	0.077	0.116	-0.055 (-0.112-0.004)
		3	Agree	22.29							
		2	Undecided	0.06							
		1	Disagree	0.03							
		0	Strongly disagree	6.77							
4	The videoconferencing system was easy to use (Patient *n* = 2104; Carer *n* = 154)										
		4	Strongly agree	72.76	4	1 [3–4]	4	1 [3–4]	**< 0.001**	**0.004**	0.117 (0.037-0.2)
		3	Agree	18.42							
		2	Undecided	0.35							
		1	Disagree	0							
		0	Strongly disagree	8.46							
5	Virtual care made it easier for me to get treatment (Patient *n* = 2896; Carer *n* = 224)										
		4	Strongly agree	65.93	4	1 [3–4]	4	1 [3–4]	**0.008**	**0.014**	-0.047 (-0.11-0.021)
		3	Agree	25.67							
		2	Undecided	0.13							
		1	Disagree	8.27							
		0	Strongly disagree	0							
6	The information given to me about Sydney Virtual was useful (Patient *n* = 2910; Carer *n* = 224)										
		4	Strongly agree	74.31	4	0 [4–4]	4	1 [3–4]	**0.008**	**0.014**	-0.047 (-0.102-0.013)
		3	Agree	21.35							
		2	Undecided	0							
		1	Disagree	4.28							
		0	Strongly disagree	0.06							
7	How would you rate the waiting time before the Care Centre clinician answered your call? (Patient *n* = 2702; Carer *n* = 201)										
		4	Excellent	2.82	3	0 [3–3]	3	0 [3–3]	**< 0.001**	**0.004**	-0.066 (-0.124–0.009)
		3	Good	78.19							
		2	Fair	0.10							
		1	Poor	10.37							
		0	Very Poor	8.51							
8	The virtual hospital clinicians explained things in a way I could understand (Patient *n* = 3024; Carer *n* = 233)										
		4	Strongly agree	84.59	4	0 [4–4]	4	0 [4–4]	0.274	0.302	-0.027 (-0.073-0.02)
		3	Agree	11.58							
		2	Undecided	0.03							
		1	Disagree	0							
		0	Strongly disagree	3.81							
9	My family/carer and I were involved as much as I wanted in making decisions about my condition and/or care needs (Patient *n* = 3019; Carer *n* = 232)										
		4	Always	79.84	4	1 [3–4]	4	1 [3–4]	**0.008**	**0.014**	-0.091 (-0.15–0.025)
		3	Mostly	0.78							
		2	Sometimes	13.71							
		1	Rarely	0.03							
		0	Never	5.64							
10	Was the information given to you about the health devices (oximeter and/or temperature patch) useful? (Patient *n* = 2033; Carer *n* = 195)										
		4	Yes, definitely	83.98	4	0 [4–4]	4	0 [4–4]	0.641	0.641	0.013 (-0.041-0.07)
		3	Yes, to some extent	12.84							
		2	No	1.44							
		1	Don’t know / can’t remember	0.63							
		0	I did not receive any information about the health devices	1.12							
11	The health devices (oximeter and/or temperature patch) were easy to use (Patient *n* = 2030; Carer *n* = 193)										
		4	Yes, definitely	87.85	4	0 [4–4]	4	0 [4–4]	**0.002**	**0.006**	0.076 (0.019–0.135)
		3	Yes, to some extent	9.85							
		2	No	1.21							
		1	Don’t know / can’t remember	1.08							
12	I felt confident in the safety of virtual treatment and care (Patient *n* = 2999; Carer *n* = 230)										
		4	Strongly agree	77.95	4	0 [4–4]	4	0 [4–4]	0.277	0.302	-0.031 (-0.081-0.021)
		3	Agree	14.37							
		2	Undecided	0.06							
		1	Disagree	7.59							
		0	Strongly disagree	0.03							

^1^Note: P value was derived using Mann-Whitney U test to compare the distribution of scores of patients and carers

Null hypothesis = The distribution of question responses is the same across carers and patients

Statistical difference level = 0.05

Benjamini–Hochberg FDR correction (α = 0.05)

Cliffs Delta interpretation: negative Cliffs Delta means Carers tends to score slightly higher than Patients

#### Likert scale questions with statistically significant differences between carers and patients

**Fig. 1:**
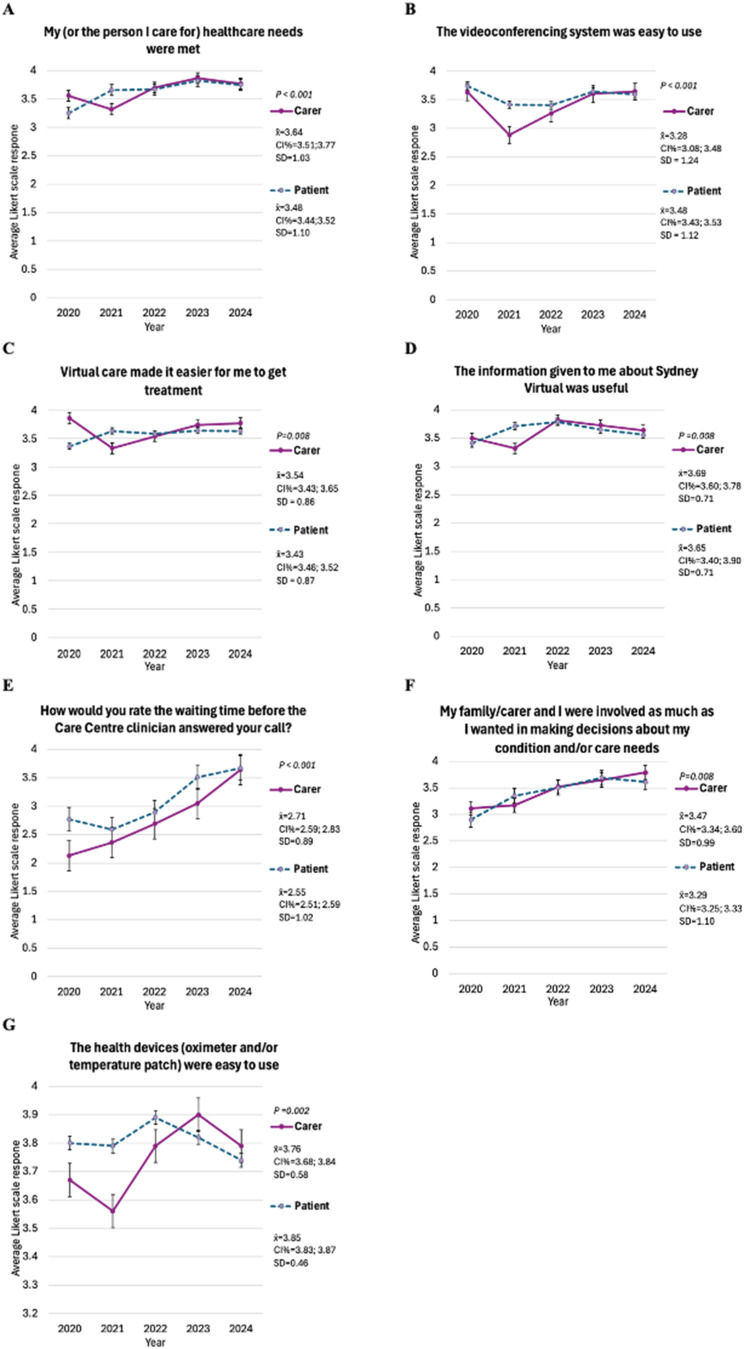
Average Likert responses over time for questions with significant differences between patients and carers. P value calculated with Mann-Whitney U test

Figure 1 presents Likert scale questions where carers and patients reported statistically significant different experiences based on the overall average. The graphs illustrate average scores for carers and patients across three years (2020–2024), allowing comparison over time. This helps visualise how experiences have evolved and improved as the model has matured (e.g., during and post-COVID-19 pandemic with large fluctuations of admission numbers). Statistically significant differences highlight areas that may matter more to carers or patients, offering insights into priorities for improving virtual hospital care.

- For Question 2, *‘My (or the person I care for) healthcare needs were met’* (Fig. 1A), carers reported a higher average Likert score (AVG = 3.64, SD = 1.03, CI% [3.51;3.77]), compared to patients (AVG = 3.48, SD = 1.10, CI% [3.44;3.52]) (*p* < 0.001). Both patients and carers show improvement over time, particularly from 2020 to 2021.
- For Question 4, *‘The videoconferencing system was easy to use’* (Fig. 1B), carers reported a lower average Likert score (AVG = 3.28, SD = 1.24, CI% [3.08; 3.48]) compared to patients (AVG = 3.48, SD = 1.12, CI% [3.43; 3.53]) (*p* < 0.001). Carer responses improve from 2021 to 2023.
- For Question 5, *‘Virtual care made it easier for me to get treatment’* (Fig. 1C), carers reported a higher average Likert score (AVG = 3.54, SD = 0.86, CI% [3.43; 3.65]) compared to patients (AVG = 3.49, SD = 0.87CI% [3.46; 3.52]) (*p* = 0.008). Both patients and carers show improvement over time. The standard deviations are small, indicating consistent responses.
- For Question 6, *‘The information given to me about Sydney Virtual was useful’* (Fig. 1D), carers reported a higher average Likert score (AVG = 3.69, SD = 0.71, CI% [3.60; 3.78]) compared to patients (AVG = 3.65, SD = 0.70, [CI%=3.40; 3.90]). The standard deviations are small, indicating consistent responses.
- For Question 7, *‘How would you rate the waiting time before the Care Centre clinician answered your call?’* (Fig. 1E), carers reported a higher average Likert score (AVG = 2.71, SD = 0.89, CI% [2.59; 2.83]) compared to patients (AVG = 2.55, SD = 1.02, CI% [2.51; 2.59]) (*p* < 0.001). Both patients and carers show improvement over time, though carers feedback demonstrated a notable, steady improvement from 2020 to 2024.
- For Question 9, *‘My family/carer and I were involved as much as I wanted in making decisions about my condition and/or care needs’*(Fig. 1F), carers reported a higher average Likert score (AVG = 3.47, SD = 0.99, CI% [3.34; 3.60]) compared to patients (AVG = 3.29, SD = 1.10, CI% [3.25; 3.33]) (*p* = 0.008). Both carers and patients show improvement over time.
- For Question 11, *‘The health devices (oximeter and/or temperature patch) were easy to use’* (Fig. 1G), carers reported a lower average Likert score (AVG = 3.76, SD = 0.58, 95% CI [3.68, 3.84]) compared to patients (AVG = 3.85, SD = 0.46, 95% CI [3.83, 3.87]) (*p* = 0.002). Both groups show improvement over time.

#### Likert scale questions with no statistically significant difference between patients and carers

**Fig. 2:**
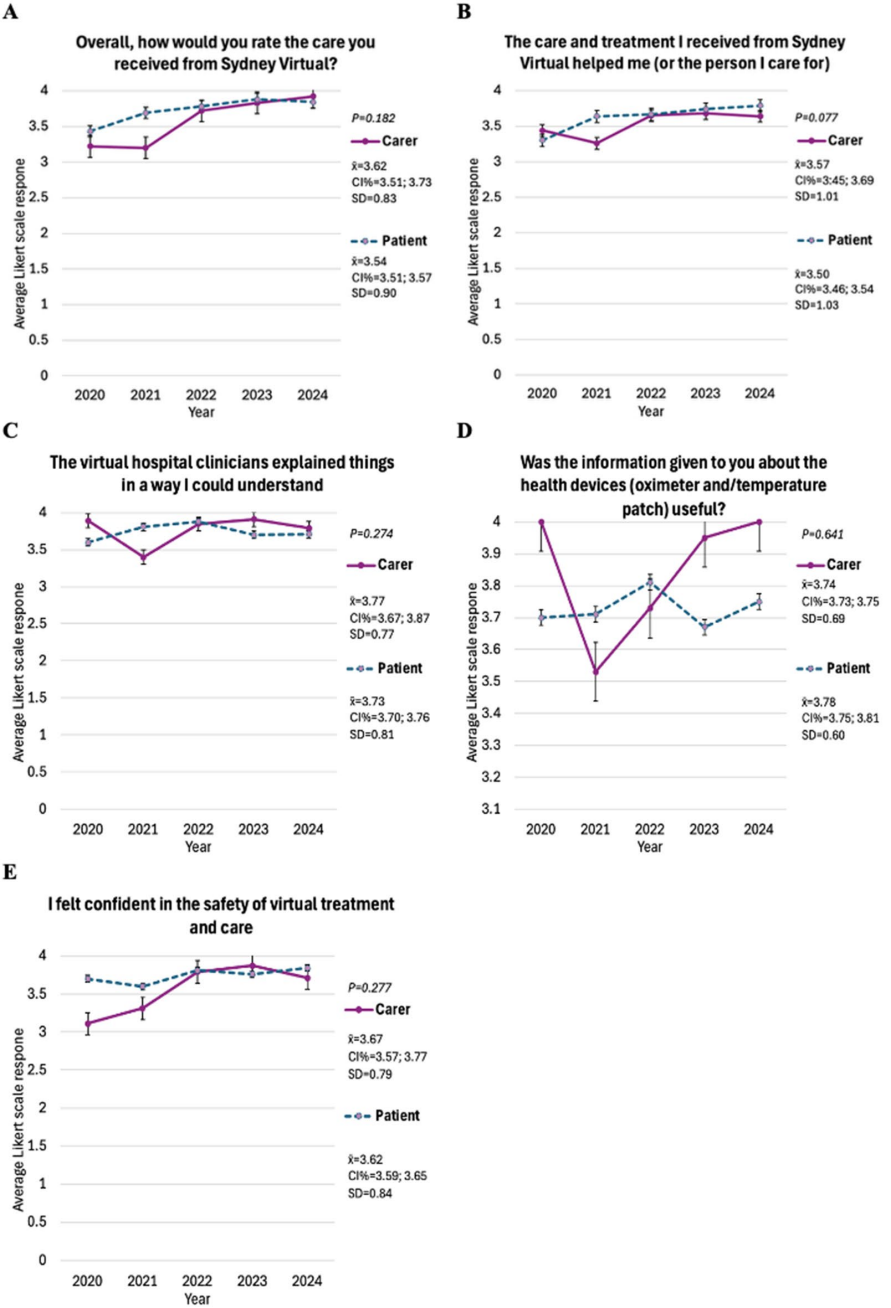
Average Likert responses over time for questions with no significant differences between patients and carers. P value calculated with Mann-Whitney U test

No significant statistical differences were found for questions 1, 3, 8, 10, and 12 indicating patient and carer perspectives may align. Figure 2 presents a graph for corresponding questions.

#### Content analysis of free-text responses of carers

**Fig. 3:**
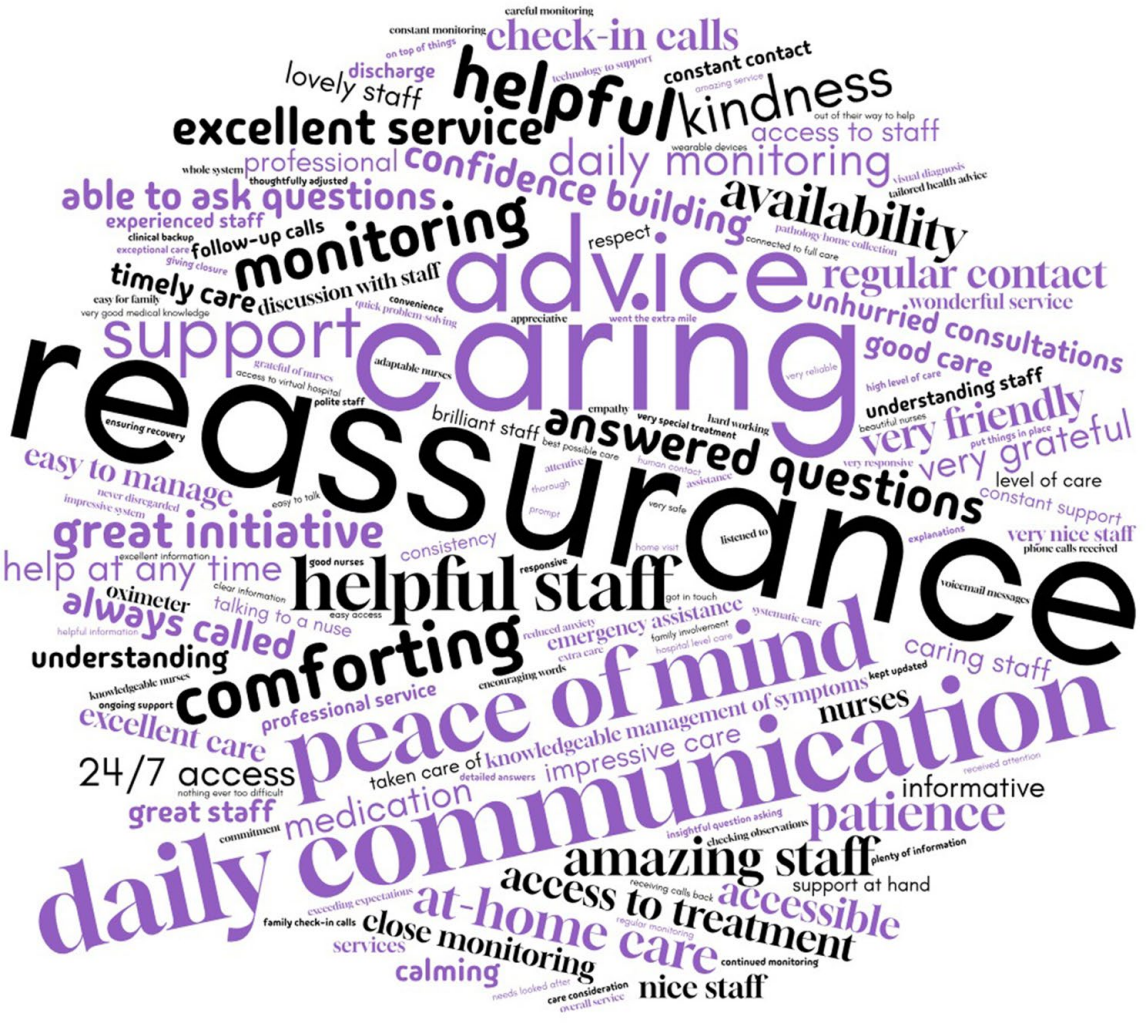
“What was the best part of the care you received from Sydney Virtual?” word cloud (*n* = 173/235). The font size reflects its frequency of occurrence in the source text (e.g., reassurance is the most frequently appearing word or phrase)

**Fig. 4:**
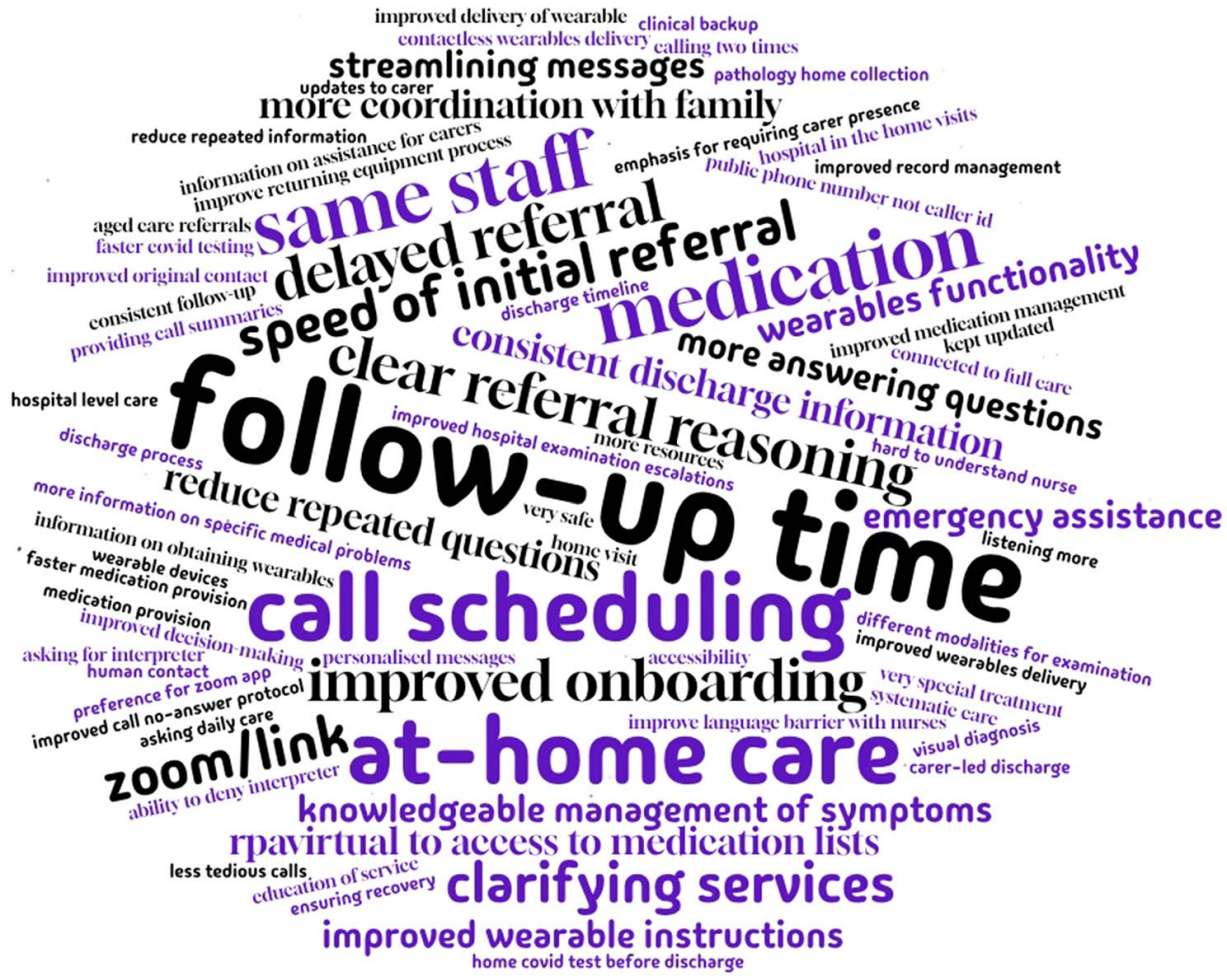
“What part of your care provided by Sydney Virtual most needs improving?” word cloud (*n* = 150/235). The font size reflects its frequency of occurrence in the source text (e.g., follow-up time is the most frequently appearing word or phrase)

Figures 3 and 4 are word clouds of 235 carers for the free-text questions (What was the best part of the care you received from Sydney Virtual? What part of your care provided by Sydney Virtual most needs improving?) as a visual representation of the rate and occurrence of words or phrases.

### Insights into improving patient and carer experience from carers’ perspective

Keywords from the content analysis of free-text responses were integrated with statistical findings from Likert-scale questions to develop overarching themes, which served as insights. We identified six key insights that improve patient and carer experience:

1. *Reassurance*: Providing comfort and confidence to patients and carers, helping reduce anxiety and build trust in the virtual hospital. Particularly important as carers adapt to new roles introduced by virtual caregiving.
2. *Effective communication*: Clear, empathetic, and inclusive information-sharing between healthcare workers, patients, and carers. Particularly important to enable shared understanding and decision-making that considers carers.
3. *Consistent availability*: Timely and accessible support (e.g., phone, email, text), with escalation pathways when needed. Particularly important for enabling carer inquiries in times of uncertainty.
4. *Supportive healthcare workers*: Professionalism, compassion, and responsiveness of clinicians. Particularly valued by carers when healthcare workers demonstrated person-centred approaches.
5. *Person-centred clinical care*: Providing clinical care and treatment, depending on the patient’s holistic needs and preferences informed with assistance from carers.
6. *Usable technology*: Tools such as remote monitoring devices and telehealth platforms are valued when easy to use, with carers highlighting the importance of clear guidance and accessible technical support.

Table 3 presents insights that reflect areas carers identified as important in their free-text responses, themes emerging from inductive content analysis, and patterns observed in relevant Likert-scale items. These were interpreted to inform data-driven improvement opportunities. While these do not constitute prescriptive recommendations, they offer structured service insights into what carers valued most or identified as areas needing improvement.

**Table 3:** Service insights proposed for improved patient and carer experiences based on statistical analysis and content analysis of carer insights (*n* = 235)

#	Primary theme	Content analysis (key word)	Free-text response example quotes (carers only)	Likert scale question responses (235 carers and 3047 patients)	Service insights derived from carers’ feedback^1^
1	Reassurance	• Reassuring (*n* = 82) • Support (*n* = 16) • Family (*n* = 7)	*Best part of care*: • “It was reassuring that a health professional was monitoring mum and checking in everyday” • “The ongoing support for my mother and the ability to ask questions. The whole process was professional” • “Best possible care… If they saw the patient is in distress they will take the time to put them at ease and comfort.”	-	• Use caring language and spend time with patients/carers answering questions to build trust, address concerns and ease uncertainty with carers. • Offer regular, personalised updates and practical advice/guidance to reassure carers about patient progress and care plans. • Identify patients who live alone and proactively engage carers (with patient consent) to reassure them that support is in place.
2	Effective communication	• Advice (*n* = 30) • Communication (*n* = 16) • Consistency (*n* = 9) • Information (*n* = 9) • Clarity (*n* = 7) • Calls (*n* = 5) • Interpreter (*n* = 2)	*Best part of care*: • “The follow up calls and ensuring recovery” • “Daily calls to monitor my young baby and providing valued and trusted advice to assist in his recovery” *Areas to improve*: • “Original contact…I didn’t know what rpavirtual (Sydney Virtual) was and was hesitant” • “Not having a clear expectation for release… which was frustrating.” • “Perhaps to ask the patient if they need an interpreter”	• Carers rated the usefulness of information higher (3.69) than patients (3.65) (*p* = 0.008). This suggests carers may value clear information to support their role. • No statistical difference was found in how clearly clinicians explain things (*p* = 0.274), indicating similar reported experiences of patients and carers. • Involvement in decision-making was rated higher by carers (3.47) than patients (3.29) (*p* = 0.008). This highlights carers value being included.	• Clearly communicate the reasons behind care decisions, as carers value understanding the “why” to feel involved and reassured. • Justify and communicate the use of interpreter services to prevent potential conflicts between patients and carers. • Streamline clear communications to reduce burden on carers, including reminders, pre-scheduled calls, and practical guides for virtual caregiving (e.g., at-home exercises, diet, pain management, and expected length of admission).
3	Consistent availability	• Regular contact (*n* = 31) • Available (*n* = 24) • Scheduling (*n* = 9) • Ease (*n* = 8) • Personal (*n* = 6) • Quick (*n* = 3)	*Best part of care*: • “Whenever I called, they were available to talk” • “The regularity of the contact. The unhurried consultations.” • “Being involved although I live in Queensland” • “Easy access. Helpful information.” • “The patient was at home and easy for family to help” *Areas to improve*: • “It would be ideal to schedule calls and appointments if possible.” • “Care delivered was focused on my child, but I was also very unwell and because calls were unscheduled they often woke me.”	• Carers rated ease-to-get treatment higher (3.54) than patients (3.49) (*p* = 0.008). This suggests carers may value the access to treatment provided by virtual hospitals. • Carers reported satisfaction with wait times higher on average (2.71) compared to patients (2.55) (*p* < 0.001). This may indicate carers appreciate the availability of the service in comparison to traditional ED of GP services wait times. Carers may appreciate an at-home setting.	• Availability of daily 24-hour support with routine and after-hours contact options for timely assistance for patients and carers. • Coordinate scheduling with both patients and carers to maximise convenience and ensure carers are available to support. • Respond promptly to queries and callbacks to maintain carer trust and reduce stress during care delivery.
4	Supportive healthcare worker	• Staff, nurses and doctors (or related interchangeable roles) (*n* = 69)	*Best part of care*: • “Communicating with the nurses and receiving call back from doctors” • “The doctors and nurses were very caring and provided lots of info” • “The staff I spoke to were very responsive, and went out of their way to answer all of my questions and provided me with information that I could then support my sister” *Areas to improve*: • “If possible to have the same nurses and doctor available at the time of consultation.”	-	• Human interactions remain essential in virtual hospitals, value of healthcare workers as helpful, kind, and patient. • Consistency in healthcare workers allocation (e.g., same nurse and same doctors where able).
5	Person-centred clinical service	• Monitoring (*n* = 20) • Referral/Onboarding (*n* = 10) • Pharmacy / medication (*n* = 8) • Discharge (*n* = 7) • Escalation (*n* = 4)	*Best part of care*: • “Great experience and was reason my elderly mother recovered much quicker” • “Medication & health device received within 24 hours” • “Organised to get me the antiviral medicine.” • “Monitoring symptoms and organised ambulance when needed” • “Felt very safe and confident we were doing everything to get a good result” *Areas to improve*: • “No one explained how or why my son was part of rpavirtual (Sydney Virtual) (from referral); the first call was totally out of the blue!”	• No significant difference was found in perceptions of whether virtual care was helpful (*p* = 0.077). Carers rated it slightly higher (3.57) than patients (3.50). • Carers rated their patients’ healthcare needs as better met higher (3.64) than patients (3.48) (*p* < 001), possibly due to appreciation of clinical monitoring. • Confidence in the safety of the virtual hospital showed no significant difference (*p* = 0.277), with similar high ratings from carers (3.67) and patients (3.62).	• Conduct regular clinical check-ins and remote examinations, providing carers with confidence that patients are closely observed. • Coordinate medications with patients, carers’, and pharmacy for safe medication management. • Establish and communicate escalation pathways to help carers understand when and how to seek urgent care, preventing avoidable or delayed ED visits. • Include carers in the referral and onboarding process to communicate expectations of technology checks, and care transitions (e.g., from GP or ED). • Include carers in discharge processes (e.g., coordinating wearable returns, connection to community GP).
6	Usable technology	• Technology / wearables (*n* = 17)	*Best part of care*: • “Knowledgeable management with technology to support” *Areas to improve*: • “Due to invalid reading an ambulance was sent to my home” • “Include that hands need to be warm to get accurate reading (on oximeter). Sometimes the zoom link wasn’t received straight away due to technical glitch.”	• For ‘The health devices (oximeter and/or temperature patch) were easy to use’ patients rated the technology as easier to use (3.85) than carers (3.76) (p = 0.002), suggesting carers may struggle more when assisting with technology.	• Checking technology functionality during onboarding and involve carers in setup (e.g., wearables functionality, Zoom/Microsoft teams link). • Investment into reliable devices • Provide ongoing technical assistance (e.g., clear troubleshooting resources and ensure staff can offer basic IT support, considering varying levels of digital literacy).

Note: ^1^Supplementary Material Table 4 outlines examples of carers’ PREMs free-text responses that contribute to these Service Insights Recommendations

## Discussion

### Key findings

**Fig. 5:**
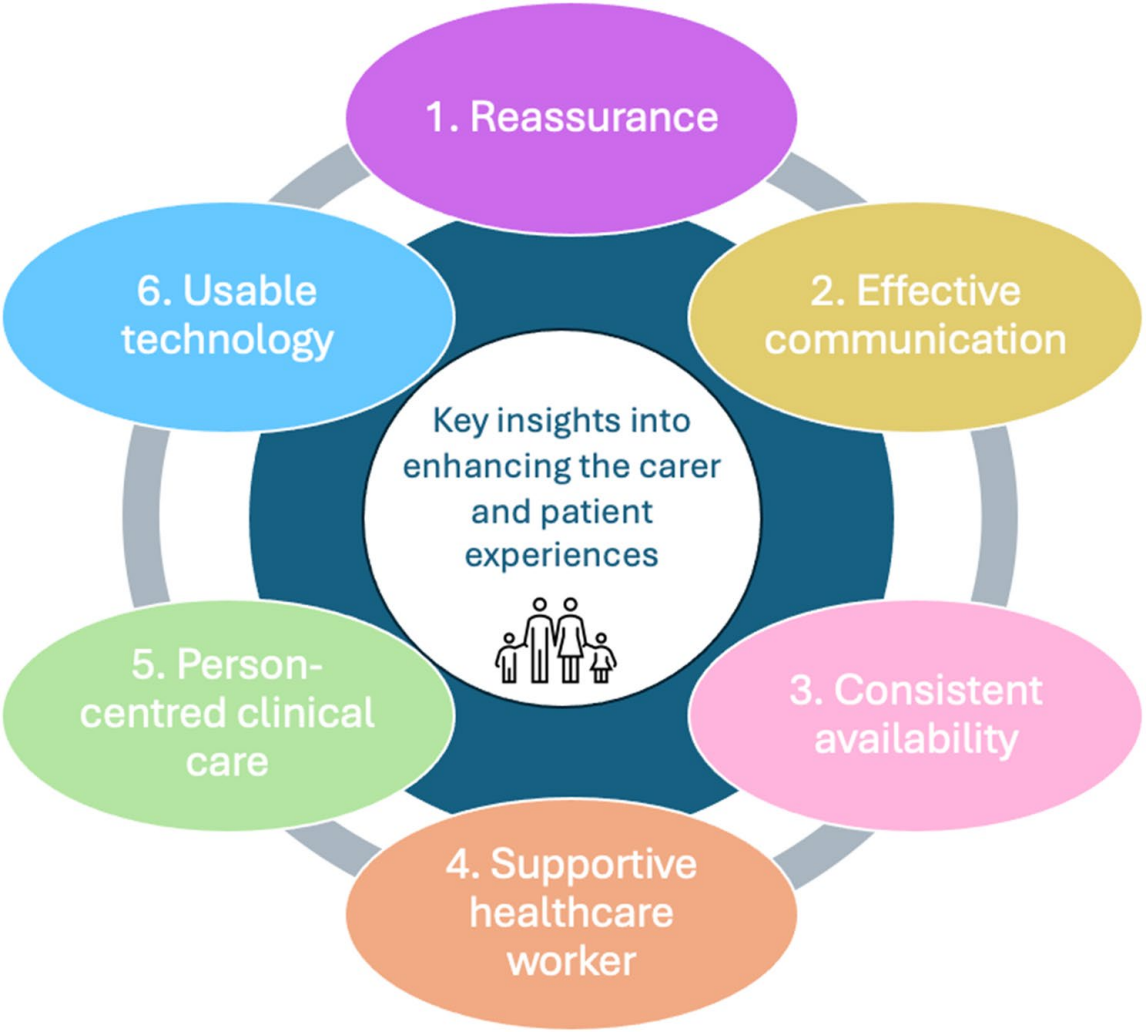
Key insights into enhancing the carer and patient experiences informed by carer’s reported experiences

This study presents PREM findings from 235 carers and 3047 patients who accessed Sydney Virtuals’ Acute Respiratory model of care, with a focus on the carer perspective. Overall, reported experiences were consistently positive. The pattern of statistically significant differences remained unchanged after FDR correction, increasing confidence that these represent true differences rather than chance findings. The most notable year-on-year variation occurred between 2020 and 2022. This period aligns with the COVID-19 pandemic, rapid service expansion, increased patient volumes, new staff recruitment, and the development of referral and escalation pathways. The overall increase in reported positive experiences across various measures may reflect carers’ growing confidence and reassurance in using the virtual hospital, and/or maturation and improvement of the service with experience accrued during the rapid upscaling. It is important to note that the strategies/improvements implemented by Sydney Virtual to address the feedback was not explored within this particular study. From the data, six key insights emerged as central to positive patient and carer experiences from carers perspectives: effective communication, consistent availability, reassurance, usable technology, person-centred clinical care, and supportive healthcare workers (see Fig. 5).

The survey questions with statistical differences in carers and patients’ reported experience may suggest that carers’ perspectives on their experiences with Sydney Virtual are shaped significantly by their responsibilities in supporting care, navigating technology, and ensuring that patients’ needs are met. This could indicate that carers took on active roles in setup and ongoing support, while patients may have adopted a more passive role when care was facilitated for them. The greater burden on carers may also explain why they were more sensitive to delays in service, highlighting the need to reduce wait times through improved queue management, better clinician availability, or more efficient admission processes. It is important to note that experiences such as longer waiting times may reflect the unprecedented surge in patient volumes during the COVID-19 period. As demand eased and staffing models adapted, service responsiveness improved. These changes in context likely influenced how carers experienced and reported aspects of care delivery over time.

Carers emphasised the importance of clear, empathetic communication from healthcare workers. Findings suggest that carers rely heavily on clear instructions and accessible, trusted services to fulfil their roles effectively, reinforcing the importance of enhancing both patient and carer engagement strategies. Effective communication not only reassures carers but also can reduce anxiety during a period of heightened uncertainty. Carers expressed a need for better explanations regarding patient progress, care decisions, and expected hospital stay duration. This transparency was seen as vital for helping carers feel informed and confident in the care process.

Technology emerged as an ‘area to improve’, which may be due to the technical support role carers take on to assist patients. Commonly reported issues by carers included malfunctioning wearables, delays in Zoom links, and inadequate technological resources (e.g., poor internet access or outdated devices). Low digital literacy among some carers may exacerbate these challenges. To improve usability, carers suggested onboarding support tailored to individual digital literacy levels, investment into reliable devices, and ongoing technical assistance. Over time, several key changes were implemented at Sydney Virtual to address feedback, which resulted in a decline in reported technology feedback in later years. Sydney Virtual introduced a Digital Patient Navigator role in 2021 to support patients with technology concerns. The video-conferencing platform was also transitioned from Zoom to Microsoft Teams in 2023 in alignment with the Local Health District supported platform. Training healthcare workers on technical troubleshooting and virtual communication (i.e., ‘netiquette’) could ease similar challenges experienced by other virtual hospital models and enhance overall patient and carer experience.

Carers reported viewing Sydney Virtual as holistic and responsive, particularly for complex patients. Friendliness and kindness were frequently reported as highlights of Sydney Virtual. Despite being virtual, care delivery still relies on strong interpersonal connection to build rapport and trust. Additionally, the admissions data indicates that the virtual hospital services potentially vulnerable groups such as elderly patients and paediatric patients. For these groups, avoiding travel and receiving continuous care at home may greatly reduce burdens, particularly for carers. While carers acknowledged and appreciated the accessibility of Sydney Virtual, delays in follow-up care were reported. Improving communication through proactive communication, clarity around wait times, and appropriate escalation pathways could reduce uncertainty and enhance patient and carer experience. As shown in the graphs, earlier years feedback was dominated by technology-related concerns and communication issues, particularly during the pandemic peak. Over time, such concerns became less prominent, while feedback around care coordination and responsiveness improved. These visual trends reflect both the evolving care environment and Sydney Virtuals’ responsiveness to PREM survey data.

### Comparison to literature

The six insights identified within our study (i.e., reassurance, effective communication, consistent availability, supportive healthcare workers, person-centred clinical care, usable technology) not only support existing findings but also extend the evidence base in the context of virtual hospitals. Singh et al. [19] demonstrated how PREM surveys can be effectively used to identify areas for improvement in palliative inpatient care, supporting the methodological use of PREM survey data in our study to explore similar quality improvement opportunities.

Effective communication and reassurance were frequently mentioned as factors of positive patient and carer experiences. Kornhaber et al. [20] highlighted that trust built through therapeutic relationships is foundational to patient confidence and reassurance. Ha and Longnecker [21] identified physician communication as critical to improving patient outcomes and mitigating dissatisfaction. Keeling, Laing, and Ruyter [22] further emphasised the increasing responsibility placed on carers to manage healthcare information, particularly in virtual settings. Our study shows that carers derive reassurance not only from interactions with healthcare workers but also from clear processes, ongoing support, and responsive communication. Our findings reinforce the role of clear, empathetic communication in helping carers feel informed, valued, and confident in navigating virtual care processes.

Carer involvement and experience was also shaped by perceptions of availability and clinical care. Gledhill et al. [23] noted that family involvement improves access to care and shared decision-making, which may be particularly important when carers help manage schedules or initiate consultations. Our findings add nuance by demonstrating that carers may facilitate scheduling and availability for consultations. Our study shows that carers in virtual models often serve as observers and intermediaries, helping escalate clinical care when needed.

Lastly, healthcare workers and technology were frequently mentioned as impacting carers’ reported experience. Carers frequently commented on the expertise, empathy, and responsiveness of healthcare workers. This supports Kornhaber et al.‘s [20] findings that relationships between healthcare workers and families can enhance trust and satisfaction. Our findings also highlight how the reliability and usability of technology shape carers’ reported experience considering their role in providing technical support. These findings complement Sundaram et al.‘s [24] work on the use of PREM surveys in service improvement, suggesting technology design and training must account for both patients and their carers.

### Implications for care provision

Sydney Virtual has evolved into a more integrated model, aiming to meet the needs of patients, carers, and advancing technology. As one of the first of its kind in Australia, Sydney Virtual serves as a model for future virtual hospitals, using patient feedback to improve services and experiences. Ongoing analysis of PREM surveys is vital for identifying challenges, improving care transitions, and refining virtual hospital models. While virtual hospitals increase accessibility, they can also place additional and unfamiliar responsibilities on carers. Carer feedback is crucial to designing carer-inclusive models and tailoring support. Future research should explore adapting PREM surveys to capture carer experiences, including more open-ended and targeted questions, or carer specific surveys.

Low digital literacy and health literacy remain barriers to virtual care. Tailored onboarding, technical support, and clear guidance are key to supporting both patients and carers. Ongoing healthcare worker training in virtual communication, remote assessment, and patient education will also enhance care delivery and overall experiences. Continued research to optimise the caregiving experience in virtual hospitals and improved virtual communication techniques is required to ensure that carers receive the support and resources required.

### Strengths and limitations

A key strength of this study is the dual focus on both patient and carer experiences to provide a comprehensive understanding of areas important to consumers and their perspectives. The inclusion of a large sample size enhances the robustness of findings and allows for broader interpretation of reported experiences across the Acute Respiratory model of care virtual hospital service as the service has evolved. The combination of Likert-scale data with open-ended responses enables depth in analysis and provided key insights for utilisation by the virtual hospital. While the Likert-scale data allowed for statistical comparison and trend analysis, the qualitative responses offered contextual nuance, capturing individual perspectives and identifying factors important to carers that may not be captured in fixed-response questions. We acknowledge the limitation of brief free-text responses that limit the contextual details. However, our code triangulation concentrates around broader conceptual categories.

There are, however, limitations to consider. While Likert-scale surveys facilitate ease of response and support quantitative analysis, they are susceptible to social desirability bias. Respondents may provide answers they perceive as socially acceptable rather than fully accurate. This tendency may be amplified in healthcare settings, where patients and carers may hesitate to criticise services, particularly if they were satisfied with clinical outcomes. Carers may also focus more on patient welfare, not their own.

A further limitation relates to survey design and completeness. Due to the nature of PREM surveys being anonymous, there is an inability to link demographic data to individual responses. This restricted our ability to describe the respondent group, assess representativeness, or adjust for case-mix differences. In addition, the response rate and voluntary nature of demographic items likely introduced some selection and non-response bias (e.g., optional items may be skipped to maintain brevity or privacy). Individuals with higher digital literacy or more positive experiences may have been more likely to respond. The survey length (36 items) may have also contributed to non-response, partial or incomplete responses.

Despite the availability of language and accessibility options, online survey distribution may have excluded individuals with low digital literacy, introducing potential selection bias. Those with negative experiences, especially related to technology or limited engagement, may have been less inclined to respond, potentially skewing results toward more positive reported experiences. The inclusion of Likert scale question formats (e.g., 3-point and 5-point Likert scales) aligns with existing PREM surveys, though it limited the comparability of responses and included questions for statistical analysis. Lastly, as PREM surveys were completed post-discharge, responses may have been affected by recall bias or if impressions were shaped more by discharge experiences.

Future iterations of the PREM survey could consider introducing a minimal set of mandatory demographic fields (e.g., age band, gender) to enable representativeness checks and enhance interpretation of findings. Given the wide age and cultural variability observed in the EMR dataset, it is reasonable to assume that the PREM respondents reflect a similarly diverse population. However, this cannot be confirmed without linked demographic data.

## Conclusion

Patients and carers reported highly positive experiences with Sydney Virtual, highlighting its success and growth. Six key insights for improving patient and carer experiences based on carers’ insights emerged: reassurance, consistent availability, effective communication, person-centred clinical care, usable technology, and supportive healthcare workers. These insights can guide refinements to virtual hospitals that integrate carers reported experiences. Future research should continue leveraging PREM survey data to improve and validate these considerations, ensuring virtual hospitals meet both clinical needs and carer support requirements.

## Supplementary Information

Below is the link to the electronic supplementary material.

Supplementary Material 1

Supplementary Material 2

## Data Availability

The data supporting this study are not publicly available due to ethical concerns regarding participant privacy and is not available upon request due to ethical approvals.

## Abbreviations

AVG: Average Response
ED: Emergency Department
GP: General Practitioner
MED: Median
PREM: Patient Reported Experience Measures
SD: Standard Deviation
