# Supplementary material for: Carer and patient experiences in a virtual hospital: service insights from a mixed-methods analysis of reported experience measures
Source: J Patient Rep Outcomes. 2026 Mar 4;10:57. doi: 10.1186/s41687-026-01029-w (PMC13069058; doi:10.1186/s41687-026-01029-w)
Supplement: Supplementary file 1 — Supplementary Material 1 [file 41687_2026_1029_MOESM1_ESM.docx]

## Plain English Abstract

Carers such as family members and companions play a vital, but often under-recognised role in care by supporting patients emotionally, practically, and through communication. Their influence is especially important in virtual hospital models, where patients receive remote hospital-level care using digital technologies. However, there is limited evidence examining what carers think about virtual hospitals or how their experiences could be improved. In this study, we have explored patient-reported experience measure surveys completed by 3047 patients and 235 carers to better understand their experiences with a virtual hospital service. This study identified service insights from carers that highlight how they support patients, shape their care experience, and offer valuable feedback. We identified six key insights that improve patient and carer experience including reassurance, effective communication, consistent availability, supportive healthcare workers, person-centred clinical service, and usable technology. Findings from this study can help improve virtual hospital models to be more inclusive and centred around both patients and carers, using data that is already being collected as part of standard care.
