# Supplementary material for: Carer and patient experiences in a virtual hospital: service insights from a mixed-methods analysis of reported experience measures
Source: J Patient Rep Outcomes. 2026 Mar 4;10:57. doi: 10.1186/s41687-026-01029-w (PMC13069058; doi:10.1186/s41687-026-01029-w)
Supplement: Supplementary file 2 — Supplementary Material 2 [file 41687_2026_1029_MOESM2_ESM.docx]

# Supplementary Material 1

**Supplementary Table S1.** Stratified PREM results by time period of Carers (n=235) and Patients (n=3047)

| **#** | **Question** | **Score** | **Text** | **%** | **Carer** | | | | | **Patient** | | | | |
| --- | --- | --- | --- | --- | --- | --- | --- | --- | --- | --- | --- | --- | --- | --- |
|  |  |  |  |  | **2020**  **AVG, MED [IQR]** | **2021**  **AVG, MED [IQR]** | **2022**  **AVG, MED [IQR]** | **2023**  **AVG, MED [IQR]** | **2024**  **AVG, MED [IQR]** | **2020**  **AVG, MED [IQR]** | **2021**  **AVG, MED [IQR]** | **2022**  **AVG, MED [IQR]** | **2023**  **AVG, MED [IQR]** | **2024**  **AVG, MED [IQR]** |
| 1 | Overall, how would you rate the care you received from Sydney Virtual? | | | | | | | | | | | | | |
|  |  | 4 | Excellent | 72.13 | AVG 3.22; MED 3 [3,4] | AVG 3.20; MED 4 [3,4] | AVG 3.72; MED 4 [4,4] | AVG 3.83; MED 4 [4,4] | AVG 3.92; MED 4 [4,4] | AVG 3.50; MED 4 [3,4] | AVG 3.32; MED 4 [3,4] | AVG 3.69; MED 4 [4,4] | AVG 3.89; MED 4 [4,4] | AVG 3.84; MED 4 [4,4] |
|  |  | 3 | Good | 18.42 |  |  |  |  |  |  |  |  |  |  |
|  |  | 2 | Fair | 4.51 |  |  |  |  |  |  |  |  |  |  |
|  |  | 1 | Poor | 2.12 |  |  |  |  |  |  |  |  |  |  |
|  |  | 0 | Very Poor | 2.82 |  |  |  |  |  |  |  |  |  |  |
| 2 | My (or the person I care for) healthcare needs were met) | | | | | | | | | | | | | |
|  |  | 4 | Always | 79.84 | AVG 3.56; MED 4 [4,4] | AVG 3.32; MED 4 [4,4] | AVG 3.70; MED 4 [4,4] | AVG 3.87; MED 4 [4,4] | AVG 3.77; MED 4 [4,4] | AVG 3.35; MED 4 [2,4] | AVG 3.36; MED 4 [4,4] | AVG 3.64; MED 4 [4,4] | AVG 3.81; MED 4 [4,4] | AVG 3.75; MED 4 [4,4] |
|  |  | 3 | Mostly | 0.78 |  |  |  |  |  |  |  |  |  |  |
|  |  | 2 | Sometimes | 13.71 |  |  |  |  |  |  |  |  |  |  |
|  |  | 1 | Rarely | 0.03 |  |  |  |  |  |  |  |  |  |  |
|  |  | 0 | Never | 5.64 |  |  |  |  |  |  |  |  |  |  |
| 3 | The care and treatment I received from **rpa**virtual helped me (or the person I care for) | | | | | | | | | | | | | |
|  |  | 4 | Strongly agree | 70.85 | AVG 3.44; MED 4 [4,4] | AVG 3.26; MED 4 [3,4] | AVG 3.65; MED 4 [4,4] | AVG 3.68; MED 4 [4,4] | AVG 3.64; MED 4 [4,4] | AVG 3.38; MED 4 [3,4] | AVG 3.29; MED 4 [3,4] | AVG 3.61; MED 4 [3,4] | AVG 3.75; MED 4 [4,4] | AVG 3.79; MED 4 [4,4] |
|  |  | 3 | Agree | 22.29 |  |  |  |  |  |  |  |  |  |  |
|  |  | 2 | Undecided | 0.06 |  |  |  |  |  |  |  |  |  |  |
|  |  | 1 | Disagree | 0.03 |  |  |  |  |  |  |  |  |  |  |
|  |  | 0 | Strongly disagree | 6.77 |  |  |  |  |  |  |  |  |  |  |
| 4 | The videoconferencing system was easy to use | | | | | | | | | | | | | |
|  |  | 4 | Strongly agree | 72.76 | AVG 3.63; MED 4 [3,4] | AVG 2.88; MED 4 [3,4] | AVG 3.26; MED 4 [3,4] | AVG 3.60; MED 4 [3.75, 4] | AVG 3.64; MED 4 [3.25, 4] | AVG 3.67; MED 4 [4,4] | AVG 3.73; MED 4 [4,4] | AVG 3.49; MED 4 [4,4] | AVG 3.60; MED 4 [3,4] | AVG 3.59; MED 4 [3,4] |
|  |  | 3 | Agree | 18.42 |  |  |  |  |  |  |  |  |  |  |
|  |  | 2 | Undecided | 0.35 |  |  |  |  |  |  |  |  |  |  |
|  |  | 1 | Disagree | 0 |  |  |  |  |  |  |  |  |  |  |
|  |  | 0 | Strongly disagree | 8.46 |  |  |  |  |  |  |  |  |  |  |
| 5 | Virtual care made it easier for me to get treatment | | | | | | | | | | | | | |
|  |  | 4 | Strongly agree | 65.93 | AVG 3.86; MED 4 [4,4] | AVG 3.33; MED 4 [3,4] | AVG 3.54; MED 4 [3,4] | AVG 3.74; MED 4 [4,4] | AVG 3.77; MED 4 [4,4] | AVG 3.37; MED 4 [3,4] | AVG 3.35; MED 4 [3,4] | AVG 3.53; MED 4 [3,4] | AVG 3.67; MED 4 [3,4] | AVG 3.62; MED 4 [3,4] |
|  |  | 3 | Agree | 25.67 |  |  |  |  |  |  |  |  |  |  |
|  |  | 2 | Undecided | 0.13 |  |  |  |  |  |  |  |  |  |  |
|  |  | 1 | Disagree | 8.27 |  |  |  |  |  |  |  |  |  |  |
|  |  | 0 | Strongly disagree | 0 |  |  |  |  |  |  |  |  |  |  |
| 6 | The information given to me about Sydney Virtual was useful | | | | | | | | | | | | | |
|  |  | 4 | Strongly agree | 74.31 | AVG 3.50; MED 3.5  [3,4] | AVG 3.32; MED 4 [3,4] | AVG 3.82; MED 4 [4,4] | AVG 3.73; MED 4 [4,4] | AVG 3.64; MED 4 [3,4] | AVG 3.45; MED 4 [3,4] | AVG 3.50; MED 4 [3,4] | AVG 3.77; MED 4 [4,4] | AVG 3.66; MED 4 [3,4] | AVG 3.56; MED 4 [3,4] |
|  |  | 3 | Agree | 21.35 |  |  |  |  |  |  |  |  |  |  |
|  |  | 2 | Undecided | 0 |  |  |  |  |  |  |  |  |  |  |
|  |  | 1 | Disagree | 4.28 |  |  |  |  |  |  |  |  |  |  |
|  |  | 0 | Strongly disagree | 0.06 |  |  |  |  |  |  |  |  |  |  |
| 7 | How would you rate the waiting time before the Care Centre clinician answered your call? | | | | | | | | | | | | | |
|  |  | 4 | Excellent | 2.82 | AVG 2.13; MED 3  [1, 3] | AVG 2.36; MED 3 [1,3] | AVG 2.69; MED 3 [3,3] | AVG 3.05; MED 3 [3,3] | AVG 3.64; MED 4 [3.25, 4] | AVG 2.67; MED 3 [3,3] | AVG 2.52; MED 3 [3,3] | AVG 2.71; MED 3 [3,3] | AVG 3.36; MED 3 [3,4] | AVG 3.67; MED 4 [3,4] |
|  |  | 3 | Good | 78.19 |  |  |  |  |  |  |  |  |  |  |
|  |  | 2 | Fair | 0.10 |  |  |  |  |  |  |  |  |  |  |
|  |  | 1 | Poor | 10.37 |  |  |  |  |  |  |  |  |  |  |
|  |  | 0 | Very Poor | 8.51 |  |  |  |  |  |  |  |  |  |  |
| 8 | The virtual hospital clinicians explained things in a way I could understand | | | | | | | | | | | | | |
|  |  | 4 | Strongly agree | 84.59 | AVG 3.89; MED 4 [4,4] | AVG 3.40; MED 4 [3,4] | AVG 3.85, MED 4 [4,4] | AVG 3.91; MED 4 [4,4] | AVG 3.79; MED 4 [4,4] | AVG 3.52; MED 4 [3,4] | AVG 3.49; MED 4 [4,4] | AVG 3.84; MED 4 [4,4] | AVG 3.75; MED 4 [4,4] | AVG 3.71; MED 4 [3,4] |
|  |  | 3 | Agree | 11.58 |  |  |  |  |  |  |  |  |  |  |
|  |  | 2 | Undecided | 0.03 |  |  |  |  |  |  |  |  |  |  |
|  |  | 1 | Disagree | 0 |  |  |  |  |  |  |  |  |  |  |
|  |  | 0 | Strongly disagree | 3.81 |  |  |  |  |  |  |  |  |  |  |
| 9 | My family/carer and I were involved as much as I wanted in making decisions about my condition and/or care needs | | | | | | | | | | | | | |
|  |  | 4 | Always | 79.84 | AVG 3.11; MED 3 [3,4] | AVG 3.17; MED 4 [3,4] | AVG 3.70; MED 4 [4,4] | AVG 3.65; MED 4 [4,4] | AVG 3.79; MED 4 [4,4] | AVG 2.92; MED 3 [2,4] | AVG 2.74; MED 4 [2,4] | AVG 3.53; MED 4 [3,4] | AVG 3.70; MED 4 [3,4] | AVG 3.61; MED 4 [3,4] |
|  |  | 3 | Mostly | 0.78 |  |  |  |  |  |  |  |  |  |  |
|  |  | 2 | Sometimes | 13.71 |  |  |  |  |  |  |  |  |  |  |
|  |  | 1 | Rarely | 0.03 |  |  |  |  |  |  |  |  |  |  |
|  |  | 0 | Never | 5.64 |  |  |  |  |  |  |  |  |  |  |
| 10 | Was the information given to you about the health devices (oximeter and/or temperature patch) useful? | | | | | | | | | | | | | |
|  |  | 4 | Yes, definitely | 83.98 | AVG 4.00; MED 4  [4, 4] | AVG 3.53; MED 4 [3,4] | AVG 3.73; MED 4 [4,4] | AVG 3.95; MED 4 [4,4] | AVG 4.00; MED 4 [4,4] | AVG 3.70; MED 4 [4,4] | AVG 3.43; MED 4 [3,4] | AVG 3.82; MED 4 [4,4] | AVG 3.70; MED 4 [4,4] | AVG 3.75; MED 4 [4,4] |
|  |  | 3 | Yes, to some extent | 12.84 |  |  |  |  |  |  |  |  |  |  |
|  |  | 2 | No | 1.44 |  |  |  |  |  |  |  |  |  |  |
|  |  | 1 | Don’t know / can’t remember | 0.63 |  |  |  |  |  |  |  |  |  |  |
|  |  | 0 | I did not receive any information about the health devices | 1.12 |  |  |  |  |  |  |  |  |  |  |
| 11 | The health devices (oximeter and/or temperature patch) were easy to use | | | | | | | | | | | | | |
|  |  | 4 | Yes, definitely | 87.85 | AVG 3.67; MED 4 [3.5, 4] | AVG 3.56; MED 4 [3,4] | AVG 3.79; MED 4 [4,4] | AVG 3.90; MED 4 [4,4] | AVG 3.79; MED 4 [4,4] | AVG 3.69; MED 4 [4,4] | AVG 3.64; MED 4 [4,4] | AVG 3.90; MED 4 [4,4] | AVG 3.84; MED 4 [4,4] | AVG 3.74; MED 4 [4,4] |
|  |  | 3 | Yes, to some extent | 9.85 |  |  |  |  |  |  |  |  |  |  |
|  |  | 2 | No | 1.21 |  |  |  |  |  |  |  |  |  |  |
|  |  | 1 | Don’t know / can’t remember | 1.08 |  |  |  |  |  |  |  |  |  |  |
| 12 | I felt confident in the safety of virtual treatment and care | | | | | | | | | | | | | |
|  |  | 4 | Strongly agree | 77.95 | AVG 3.11; MED 4 [3,4] | AVG 3.31; MED 4 [3,4] | AVG 3.79; MED 4 [4,4] | AVG 3.87; MED 4 [4,4] | AVG 3.71; MED 4 [4,4] | AVG 3.71; MED 4 [4,4] | AVG 3.62; MED 4 [3,4] | AVG 3.70; MED 4 [4,4] | AVG 3.81; MED 4 [4,4] | AVG 3.84; MED 4 [4,4] |
|  |  | 3 | Agree | 14.37 |  |  |  |  |  |  |  |  |  |  |
|  |  | 2 | Undecided | 0.06 |  |  |  |  |  |  |  |  |  |  |
|  |  | 1 | Disagree | 7.59 |  |  |  |  |  |  |  |  |  |  |
|  |  | 0 | Strongly disagree | 0.03 |  |  |  |  |  |  |  |  |  |  |

**Note:**

Averages are provided for descriptive interpretation of annual trends only. Inferential statistics use Median [IQR] due to the ordinal nature of Likert items.

AVG = Average

IQR = Inter Quartile Range

MED = Median

**Supplementary Table S2.** Best part of care content analysis codebook (n=173/235)

| **Category** | **Sub-category** | **Word cloud code** | **Sub-codes** | **Frequency** | **Example** |
| --- | --- | --- | --- | --- | --- |
| **Communication** | **Advice** | Advice | Valued~advice  Trusted~advice  Medical~advice | 13 | “Tips and advice about the red flags what to do etc.” – Participant ID 2350 |
|  |  | Answered~questions |  | 5 | “They were able to answer my questions.” – Participant ID 725 |
|  |  | Able~to~ask~questions |  | 4 | “Able to contact to ask questions.” – Participant ID 2981 |
|  |  | Informative | Incredibly~informative | 3 | “They were friendly and informative and allayed any fears I had and answered my questions.” – Participant ID 2656 |
|  |  | Detailed~answers |  | 1 | “Doctor answers in detail. And will follow up my questions.” – Participated 3190 |
|  |  | Tailored~health~advice |  | 1 | “Tailored health advice for my child.” – Participant 3430 |
|  | **Calls** | Phone~calls~received |  | 1 | “Surprise that there was a virtual hospital. Very impressed with the care and phone calls I received.” – Participant ID 2905 |
|  |  | Receiving~calls~back |  | 1 | “Communication with staff, receiving calls back.” |
|  |  | Talking~to~a~nuse | Nurse~calls | 2 | “Just talking to a nurse.” – Participant ID 1954 |
|  |  | Voicemail~messages |  | 1 | “… She must have been high priority. Nurses who called left messages and voicemail messages so that was convenient.” – Participant ID 3395 |
|  | **Clarity** | Clear~information | Clear | 1 | “The 1st call nurse, she can speak my language Cantonese and gave clear information. She was very patient to answer my question and made me clam.” – Participant ID 2427 |
|  | **Communication** | Insightful~question~asking |  | 1 | “... The insightful questions asked by the team.” – Particiapnt ID 3013 |
|  |  | Listened~to |  | 1 | “They listened to my concerns and treated me with respect, kindness and understanding. Never disregarded any of my concerns.” – Participant ID 3664 |
|  |  | Kept~updated |  | 1 | “Kept updated” – Participant ID 1348 |
|  | **Consistency** | Commitment |  | 1 | “Their commitment to assisting with my elderly Mum. Nothing was ever a problem or too difficult.” – Participant ID 3258 |
|  |  | Consistency |  | 2 | “Consistent care” – Participant ID 2132 |
|  |  | Very~reliable |  | 1 | “Very reliable and caring” – Participant ID 1199 |
|  |  | Very~responsive |  | 1 | “I also had direct contact with RPAVH and the staff I spoke to were very responsive and went out of their way to answer all of my questions and provided me with information that I could then support my sister to understand (to the degree she was able to) …” – Participant ID 3110 |
|  | **Discharge** | Discharge |  | 2 | “… Nurses and doctors who followed up our questions and necessary discharge information for our GP.” – Participant ID 3370 |
|  | **Information** | Helpful~Information |  | 1 | “Easy access. Helpful information.” – Participant ID 2536 |
|  |  | Excellent~information |  | 1 | “To be able to get advice and know only phone call away to help for my mum. Very supportive and without it would of made me nervous about how my mums illness was going. It put my mind at rest as the information was excellent and didn't have to leave the home” – Participant 3783 |
|  |  | Explanations | Nurse~explanations | 2 | “Mostly the explanation they did about COVID and oximeter” – Participant ID 2512  “Home visit by nursing staff to explain technology issued...” – Participant ID 3334 |
|  |  | Plenty~of~information |  | 1 | “Plenty of information and services if needed” – Participant ID 2036 |
|  |  | Very~good~medical~knowledge |  | 1 | “The medical information they knew what they were talking about. Very good medical knowledge.” – Participant ID 2089 |
| **Reassurance** | **Support** | Helpful | Very~helpful Tremendous~help | 7 | “My husband cared for me the information provided by rpa virtual was very helpful.” – Participant ID 2858 |
|  |  | Support | Great~support  Excellent~support  Supportive~process  Supported  Very~supportive | 5 | “Excellent service and support” – Participant ID 2014  “Empathy was identified in the way we were supported through the process.” – Participant ID 2796 |
|  |  | Got~in~touch |  | 1 | “[rpavirtual HCW] was really good to get in touch and put things in place for us.” – Participant ID 1336 |
|  |  | Hard~working |  | 1 | “Always rang to make sure everyone was ok thanked you so much for their concern god bless them for their hard work” – Participant ID 1452 |
|  |  | Ongoing~support |  | 1 | “The ongoing support for my mother and the ability to ask questions. The whole process was professional, and all the clinical staff were patient and caring... A BIG thank you...” – Participant ID 2623 |
|  |  | Out~of~their~way~to~help |  | 1 | “Excellent daily follow up. Wonderful service, not widely advertised/known but fantastic. Special thanks to a nurse named [rpavirtual HCW name], went out of her way to help, support my father to ensure his well-being. Pls pass on my gratitude to her.” – Participant ID 2839 |
|  | **Family** | Family~check-in~calls |  | 1 | “Calling myself & my brother to see if mum was doing.” – Participant ID 2758 |
|  |  | Family~involvement |  | 1 | “My oldest sister” – Participant ID 2088 |
|  | **Reassurance** | Caring |  | 19 | “They were very helpful and caring.” – Participant ID 2189 |
|  |  | Reassurance | Reassuring  Reassuring~staff | 27 | “It was reassuring that a health professional was monitoring mum and checking in every day to see she was ok.” – Participant ID 3213 |
|  |  | Comforting |  | 7 | “All the Doctors and Nurses were very nice and comforting and always stressed if any problems to call them ASAP.” – Participant ID 1491 |
|  |  | Peace~of~mind | Mind~at~rest  Put~mind~at~ease | 9 | “Peace of mind” – Participant ID 2819 |
|  |  | Very~grateful |  | 4 | “… I am very, very grateful to the RNs I spoke to and was happy to know they too had clinical backup as needed.” – Participant ID 3110 |
|  |  | Calming |  | 3 | “The 1st call nurse, she can speak my language Cantonese and gave clearly information. She was very patient to answer my question and made me clam.” – Participant ID 2427 |
|  |  | Confidence~building | Build~confidence | 4 | “Discussion with nursing staff and the doctor calling as soon as they could at all hours of the day as required. That was amazing. Knowing I had available support just a phone call away 24/7. As a carer of a paraplegic husband who had COVID the support gave me a lot more confidence.” – Participant ID 2802 |
|  |  | Taken~care~of |  | 2 | “Always called, taken care of.” – Participant ID 1389 |
|  |  | Appreciative |  | 1 | “RPAvirtual organised to get me the antiviral medicine on time. Really appreciated what you did all for me. My experience from rpavirual was more than expected.” – Participant ID 2367 |
|  |  | Encouraging~words |  | 1 | “When they check me on time and they give me the right words to encourage me while when I'm on a difficult condition.” – Participant ID 1741 |
|  |  | Giving~closure |  | 1 | “Giving closure to issues that had arisen.” – Participant ID 2462 |
|  |  | Never~disregarded |  | 1 | “They listened to my concerns and treated me with respect, kindness and understanding. Never disregarded any of my concerns.” – Participant ID 3664 |
|  |  | Put~things~in~place |  | 1 | “[rpavirtual hcw name] was really good to get in touch and put things in place for us.” – Participant ID 1336 |
|  |  | Reduced~anxiety |  | 1 | “Care consideration. Reduced anxiety, built confidence.” – Participant ID 3458 |
| **Availability / accessibility** | **Ease** | Easy~to~manage | Ease | 3 | “All of it, with their help it was easy to manage.” – Participant ID 3391 |
|  |  | Easy~access |  | 1 | “Easy access. Helpful information.” – Participant ID 2536 |
|  |  | Convenience |  | 1 | “… Nurses who called left messages and voicemail messages so that was convenient.” – Participant ID 3395 |
|  |  | Easy~for~family |  | 1 | “The patient was a home and easy for family to help.” – Participant ID 3309 |
|  |  | Easy~to~talk | Easy~to~understand | 1 | “How easy to talk and understand.” – Participant ID 2695 |
|  | **Personal** | Thoughtfully~adjusted |  | 1 | “Patient had intellectual disability and limited verbal communication. The nurses were very adaptable as soon as they knew this and thoughtfully adjusted how they communicated with patient (via her advocate).” - Participant ID 2239 |
|  |  | Thorough |  | 1 | “My daughter received excellent care. Were very thorough.” Participant ID 1997 |
|  |  | Understanding | Understanding~care  Understanding~needs | 3 | “The care and understanding the needs of our clients, residents, workers Thank you for your Amazing remarkable work to all.” – Participant ID 3158 |
|  | **Quick** | Prompt |  | 1 | “Prompt medical attention for a housebound patient.” – Participant 2684 |
|  |  | Quick~problem-solving |  | 1 | “I can speak to doctor virtually in such a short time. I solve the problem quickly is the best part.” – Participant ID 3777 |
|  |  | Responsive |  | 1 | “Incredibly informative, responsive and respectful. Very much appreciated overall service. Thank you.” – Participant ID 2902 |
|  | **Regular contact** | Daily~communication | Daily~calls  Daily~check-ins  Daily~contact | 11 | “The care and check in calls daily. Mum is immunocompromised and this provided us with the care she needed.” – Participant ID 2914 |
|  |  | Constant~support |  | 2 | “Constant support and reassurance from doctors and nurses.” – Participant ID 3326 |
|  |  | Daily~monitoring | Daily~nurse~contact  Daily~review  Daily~clinical~care | 5 | “Young daughter was given very special treatment, daily monitoring. She must have been high priority. Nurses who called left messages and voicemail messages so that was convenient.” – Participant ID 3395 |
|  |  | Regular~contact | Regular~checks | 4 | “Regular contact, monitoring of my health systems.” – Participant ID 2820 |
|  |  | Constant~contact | Constant~calls | 2 | “Constant contact” – Participant ID 1784 |
|  |  | Follow-up~calls | Excellent~daily~follow-up | 2 | “The follow up calls and ensuring recovery.” – Participant ID 3338 |
|  |  | Timely~care | Quickly | 3 | “I can speak to doctor virtually in such a short time. I solve the problem quickly is the best part.” – Participant ID 3777 |
|  | **Scheduling** | Unhurried~consultations | Staff~took~their~time  Stayed~for~over~an~hour | 3 | “The regularity of the contact. The unhurried consultations. The insightful questions asked by the team.” – Participant ID 3013  “The Drs and nurses were phenomenal and outstanding at RPA ICU green ward. Best possible care and can't thank them enough. If they saw the patient is in distress, they will take the time to put them at ease and comfort at the most difficult and embarrassing times. Have been in the best possible care. One in particular nurse (islander appearance) stayed with my mum for over an hour comforting her when she was struggling the most. God bless her and I can't thank her enough for looking after my mum when she was struggling.” – Participant ID 740 |
|  | **Availability** | Availability | Always~available | 5 | “Constant monitoring. Always available when needed.” – Participant ID 3325 |
|  |  | 24/7~access |  | 4 | “The nurses were very kind. it was comforting to know that there was 24-hour care available if We needed it.” – Participant 3201 |
|  |  | Always~called |  | 4 | “Always call and take care of us.” – Participant ID 1389 |
|  |  | Help~at~any~time |  | 4 | “Just knowing there was someone else there tom help at any time, or to ask.” – Participant ID 2130 |
|  |  | Support~at~hand |  | 2 | “The support at hand when needed.” – Participant ID 2343 |
|  |  | Access~to~virtual~hospital |  | 1 | “Surprise that there was a virtual hospital. Very impressed with the care and phone calls I received.” – Participant ID 2905 |
|  |  | Accessible | Accessibility  Access~to~care | 4 | “Helpful and accessible” – Participant ID 2059 |
|  |  | Assistance |  | 1 | “Assistance with care of my son” – Participant ID 341 |
| **Clinical service** | **Clinical** | At-home~care |  | 5 | “Not having to go to hospital & be looked after at home.” – Participant ID 3782 |
|  |  | Clinical~backup |  | 1 | “… I am very, very grateful to the RNs I spoke to and was happy to know they too had clinical backup as needed.” – Participant ID 3110 |
|  |  | Connected~to~full~care |  | 1 | “Felt connected to full care” – Participant ID 3321 |
|  |  | Ensuring~recovery |  | 1 | “The follow up calls and ensuring recovery.” – Participant ID 3338 |
|  |  | Home~visit |  | 1 | “Home visit by nursing staff to explain technology issued. As [patient name] does not have internet she was serviced by phone from RPAVirtual.” – Participant ID 3334 |
|  |  | Hospital~level~care |  | 1 | “Having the benefits of hospital care at home where I am comfortable and happy.” – Participant ID 2589 |
|  |  | Human~contact |  | 1 | “The human contact, kindness, and reassurance.” – Participant ID 3386 |
|  |  | Knowledgeable~management~of~symptoms | Knowledgeable~about~symptoms | 2 | “Knowledgeable management of symptoms, with technology to support.” – Participant ID 3101 |
|  |  | Systematic~care |  | 1 | “The systematic care” – Participant ID 2425 |
|  |  | Very~safe |  | 1 | “Felt very safe and confident we were doing everything to get a good result.” – Participant ID 2767 |
|  |  | Very~special~treatment |  | 1 | “Young daughter was given very special treatment, daily monitoring. She must have been high priority. Nurses who called left messages and voicemail messages so that was convenient.” – Participant ID 3395 |
|  |  | Visual~diagnosis |  | 1 | “Ability of Dr to visually diagnose patient and make decision to hospitalise patient.” – Participant ID 2849 |
|  |  | Pathology~home~collection |  | 1 | “Consistent support provided throughout the process. Assurance that you can discuss concerns with nurses and doctors. Provided pathology home collection. Nurses and doctors who followed up our questions and necessary discharge information for our GP.” – Participant ID 3370 |
|  | **Escalation** | Emergency~assistance | Ambulance | 2 | “Monitoring symptoms and organised ambulance when needed.” – Participant ID 2127 |
|  | **Pharmacy / medication** | Medication | Coordinating~pharmacy | 4 | “Medication & health device received within 24 hours after consultation.” – Participant ID 2534 |
|  | **Monitoring** | Monitoring |  | 6 | “It was reassuring that a health professional was monitoring mum and checking in every day to see she was ok.” – Participant ID 3213 |
|  |  | Check-in~calls |  | 4 | “The check ins from the nurse and consultations with the doctors.” – Participant ID 2233 |
|  |  | Close~monitoring |  | 3 | “The close monitoring of my son and myself. Knowing that help was close by if needed.” – Participant ID 986 |
|  |  | Attentive |  | 1 | “They were very attentive and stayed on top of my husband care.” – Participant ID 3619 |
|  |  | Careful~monitoring |  | 1 | “Reassurance that my child's health was being carefully monitored.” – Participant ID 3453 |
|  |  | Checking~observations |  | 1 | “Monitoring temperature. Checking observations 3 times a day over the phone.” – Participant ID 760 |
|  |  | Constant~monitoring |  | 1 | “Constant monitoring. Always available when needed.” – Participant ID 3325 |
|  |  | Continued~monitoring |  | 1 | “Continued monitoring” – Participant ID 3208 |
|  |  | Received~attention |  | 1 | “The fact that my mother received attention when we weren't expecting it. The took care of her needs and reassured her.” – Participant ID 2594 |
|  |  | Regular~monitoring |  | 1 | “Regular monitoring” – Participant ID 3146 |
|  | **Service** | Excellent~service |  | 5 | “Excellent service and support” – Participant ID 2014 |
|  |  | Access~to~treatment |  | 4 | “Treatment and care” – Participant ID 874 |
|  |  | Excellent~care |  | 3 | “My daughter received excellent care. Were very thorough.” – Participant ID 1997 |
|  |  | Support |  | 3 | “The support was comforting” – Participant ID 2547 |
|  |  | Level~of~care |  | 2 | “Level of care” – Participant ID 2652 |
|  |  | Professional~service |  | 2 | “The ongoing support for my mother and the ability to ask questions. The whole process was professional, and all the clinical staff were patient and caring... A BIG thank you...” – Participant ID 2623 |
|  |  | Services |  | 2 | “Plenty of information and services if needed.” – Participant ID 2036 |
|  |  | Amazing~service |  | 1 | “Caring Doctors and nurses checking in my baby. Amazing services I was very impressed.” – Participant ID 2682 |
|  |  | Best~possible~care |  | 1 | “… Have been in the best possible care...” – Participant ID 740 |
|  |  | Care~consideration |  | 1 | “Care consideration. Reduced anxiety-built confidence.” – Participant ID 3458 |
|  |  | Exceeding~expectations |  | 1 | “RPAvirtual organised to get me the antiviral medicine on time. Really appreciated what you did all for me. My experience from rpavirual was more than expected.” – Participant ID 2367 |
|  |  | Exceptional~care |  | 1 | “The exceptional care and understanding of ALL the nurses and doctors. They were all amazing. Friendly and professional.” – Participant ID 3791 |
|  |  | Extra~care |  | 1 | “I was not expecting the extra care so was extremely happy with it.” – Participant ID 2330 |
|  |  | Great~initiative | Impressive~initiative  Very good~initiative  Great~experience  Great~services | 5 | “The check ins throughout the day put my mind at ease as I was caring for my mother. The staff were lovely and so caring. This service was a great initiative and help.” – Participant ID 3310 |
|  |  | High~level~of~care |  | 1 | “The knowledge from the nurses and the high level of care and concerns for my parents.” – Participant ID 1178 |
|  |  | Impressive~care | Impressed | 3 | “Everything was perfect and i am very impressed with the service” – Participant ID 3314 |
|  |  | Impressive~system |  | 1 | “Level of care was unexpected, very impressed with system in place” – Participant ID 3305 |
|  |  | Needs~looked~after |  | 1 | “Knowing my needs were looked after.” – Participant ID 3582 |
|  |  | Nothing~ever~too~difficult |  | 1 | “Their commitment to assisting with my elderly Mum. Nothing was ever a problem or too difficult.” – Participant ID 3258 |
|  |  | On~top~of~things |  | 1 | “The nurses and doctors were lovely. They were on top of things. Very caring.” – Participant ID 3184 |
|  |  | Overall~service |  | 1 | “Incredibly informative, responsive and respectful. Very much appreciated overall service. Thank you.” – Participant ID 2902 |
|  |  | Very good~care | Really~good~care | 3 | “Very good, excellent.” – Participant ID 908 |
|  |  | Went~the~extra~mile |  | 1 | “The beautiful nurses always lovely and polite always went the extra mile to help my father. Thank you to all.” – Participant ID 3245 |
|  |  | Whole~system |  | 1 | “The whole system” – Participant ID 2606 |
|  |  | Wonderful~service | Wonderful | 2 | “Excellent daily follow up. Wonderful service, not widely advertised/known but fantastic. Special thanks to a nurse named [rpavirtual HCW name], went out of her way to help, support my father to ensure his well-being. Pls pass on my gratitude to her.” – Participant ID 2839 |
| **Staff** | **Staff** | Amazing~staff |  | 5 | “The exceptional care and understanding of ALL the nurses and doctors. They were all amazing. Friendly and professional.” – Participant ID 3791 |
|  |  | Helpful~staff | Helpful~nurses | 6 | “Speaking to someone friendly and helpful when I didn't understand something.” – Participant ID 3052 |
|  |  | Access~to~staff |  | 3 | “Consistent support provided throughout the process. Assurance that you can discuss concerns with nurses and doctors. Provided pathology home collection. Nurses and doctors who followed up our questions and necessary discharge information for our GP.” – Participant ID 3370 |
|  |  | Caring~staff |  | 3 | “Caring nursing staff and doctors” – Participant ID 399 |
|  |  | Great~staff |  | 3 | “Helped me so much by checking in every day. I was so worried because of my age and medical history. The nurses and doctors and social workers were just great.” – Participant ID 3154 |
|  |  | Kindness | Kind~nurses  Kind~staff | 6 | “The nurses were very kind. it was comforting to know that there was 24-hour care available if We needed it.” – Participant ID 3201 |
|  |  | Lovely~staff |  | 3 | “The nurses and doctors were lovely. They were on top of things. Very caring.” – Participant ID 3184 |
|  |  | Nurses |  | 3 | “Nurses” – Participant ID 2729 |
|  |  | Brilliant~staff |  | 2 | “The person who assisted me was brilliant. He calmed me, found out about me and my symptoms and answered all my questions with lot of patience. Everyone who contacted me were always caring and wonderful.” – Participant ID 2578 |
|  |  | Adaptable~nurses |  | 1 | “Patient had intellectual disability and limited verbal communication. The nurses were very adaptable as soon as they knew this and thoughtfully adjusted how they communicated with patient (via her advocate).” – Participant ID 2239 |
|  |  | Beautiful~nurses |  | 1 | “The beautiful nurses always lovely and polite always went the extra mile to help my father. Thankyou to all.” – Participant ID 3245 |
|  |  | Discussion~with~staff | Communication~with~staff | 2 | “Discussion with nursing staff and the doctor calling as soon as they could at all hours of the day as required. That was amazing. Knowing I had available support just a phone call away 24/7. As a carer of a paraplegic husband who had COVID the support gave me a lot more confidence.” – Participant ID 2802 |
|  |  | Empathy |  | 1 | “Empathy was identified in the way we were supported through the process.” – Participant ID 2796 |
|  |  | Experienced~staff | Highly~experienced | 2 | “Talking to someone who was experienced and concerned.” – Participant ID 2609  “Very highly experienced.” – Participant ID 2421 |
|  |  | Good~nurses |  | 1 | “Nurses were really good and understanding.” – Participant ID 433 |
|  |  | Grateful~of~nurses |  | 1 | “Excellent daily follow up. Wonderful service, not widely advertised/known but fantastic. Special thanks to a nurse named [rpavirtual HCW name], went out of her way to help, support my father to ensure his well-being. Pls pass on my gratitude to her.” – Participant ID 2839 |
|  |  | Knowledgeable~nurses |  | 1 | “The knowledge from the nurses and the high level of care and concerns for my parents.” – Participant ID 1178 |
|  |  | Nice~staff | Nice~nurses | 3 | “All the Doctors and Nurses were very nice and comforting and always stressed if any problems to call them ASAP.” – Participant ID 1491 |
|  |  | Patience | Patient~staff  Patient~nurse | 5 | “The person who assisted me was brilliant. He calmed me, found out about me and my symptoms and answered all my questions with lot of patience. Everyone who contacted me were always caring and wonderful.” – Participant ID 2578 |
|  |  | Polite~staff |  | 1 | “The beautiful nurses always lovely and polite always went the extra mile to help my father. Thankyou to all.” – Participant ID 3245 |
|  |  | Professional | Professional~staff  Highly~professional | 3 | “The exceptional care and understanding of ALL the nurses and doctors. They were all amazing. Friendly and professional.” – Participant ID 3791 |
|  |  | Respect | Respectful | 2 | “They listened to my concerns and treated me with respect, kindness and understanding. Never disregarded any of my concerns.” – Participant ID 3664 |
|  |  | Understanding~staff | Understanding~nurses | 2 | “The exceptional care and understanding of ALL the nurses and doctors. They were all amazing. Friendly and professional.” – Participant ID 3791 |
|  |  | Very~friendly | Friendly~staff | 4 | “Very friendly and excellent service received.” – Participant ID 3111 |
|  |  | Very~nice~staff | Very~nice | 2 | “All the Doctors and Nurses were very nice and comforting and always stressed if any problems to call them ASAP.” – Participant ID 1491 |
| **Technology** | **Technology** | Technology~to~support |  | 1 | “Knowledgeable management of symptoms, with technology to support.” – Participant ID 3101 |
|  |  | Oximeter |  | 2 | “Oximeter” – Participant ID 1562 |
|  |  | Wearable~devices |  | 1 | “Medication & health device received within 24 hours after consultation.” – Participant ID 2534 |

***Note:**

A single participant response can feature more than one key word or phrase.

Keywords related to nothing, or negative feedback were excluded from the best parts of care word cloud as they did not mention any best parts of care.

**Supplementary Table S3.** Areas to improve analysis codebook* (n=150/235)

| **Category** | **Sub-category** | **Word cloud code** | **Sub-codes** | **Frequency** | **Example** |
| --- | --- | --- | --- | --- | --- |
| **Communication** | **Advice** | More~information~on~specific~medical ~problems |  | 1 | “More information on what could potentially be the cause of specific medical problems, not just one solution.” – Participant ID 2462 |
|  |  | More~answering~questions |  | 2 | “Give answers to simple questions.” – Participant ID 949 |
|  | **Calls** | Less~tedious~calls |  | 1 | “Maybe calling them was a little tedious.” – Participant ID 2059 |
|  |  | Public~phone~number~not~Caller~ID |  | 1 | “I did not like the caller Id being linked to no caller ID.” – Participant ID 2697 |
|  | **Clarity** | Clarifying~services |  | 3 | “1. It took too long for initial contact to be made from when RAT registered; the most acute period of illness already over, and I had already sought telehealth privately over my concerns about my son. 2. Noone explained how or why my son was part of rpavirtual; the first call was totally out of the blue!” – Participant ID 3396 |
|  |  | Clear~referral~reasoning | Explaining~reason~for~service | 3 | “The patient was cleared of covid & discharged 3 weeks ago & is not in isolation so why does a 90-year-old woman need to do this...?” – Participant ID 887 |
|  | **Referral / Onboarding** | Delayed~referral |  | 3 | “1. It took too long for initial contact to be made from when RAT registered; the most acute period of illness already over, and I had already sought telehealth privately over my concerns about my son. 2. Noone explained how or why my son was part of rpavirtual; the first call was totally out of the blue!” – Participant ID 3396 |
|  |  | Improved~onboarding |  | 3 | “RPA virtual needs to listen before they explain something and those registration system was confusing.” – Participant ID 1018 |
|  |  | Speed~of~initial~referral |  | 3 | “Speed up the advice/ caring service” – Participant ID 2691 |
|  |  | Improved~original~contact |  | 1 | “Original contact because I didn't know what RPAVirtual was, and was hesitant to get involved.” – Participant ID 2609 |
|  | **Communication** | Hard~to~understand~nurse |  | 1 | “Sometimes could not understand the nurse. Also found that I was asked questions which were already covered by or advised to other nurses... maybe not communicating information between nurses?” – Participant ID 2130 |
|  |  | Improve~language~barrier~with~nurses |  | 1 | “Some of the nurses were a little hard to understand. We weren't always called when promised and I had to follow up. Often, we were given conflicting information when speaking with different people. Overall, very grateful for the service.” – Participant ID 1133 |
|  |  | More~communication |  | 1 | “Communication” – Participant ID 894 |
|  |  | Reduce~repeated~questions | Reduce~repeating~information | 2 | “Sometimes could not understand the nurse. Also found that I was asked questions which were already covered by or advised to other nurses... maybe not communicating information between nurses?” – Participant ID 2130 |
|  |  | Follow-up~time |  | 7 | “It appears there is a lack of resources re. Follow up time, however it was excellent under the circumstances.” – Participant ID 2796 |
|  |  | Listening~more |  |  | “Rpa virtual needs to listen before they explain something and those registration system was confusing.” – Participant ID 1018 |
|  | **Consistency** | Consistent~discharge~information |  | 2 | “I believe the information given was conflicting to what the hospital said. Patients should be asked to have a covid test before discharge.” – Participant ID 1344 |
|  |  | Streamlining~messages |  | 2 | “I received several text messages from different departments all wanting to know same information. Could the procedures be a bit more streamlined?” – Participant ID 2725 |
|  | **Discharge** | Aged~care~referrals |  | 1 | “Should have provided aged care referrals as GP not helping with housebound patient who can't get a doctor to see her at home for any checkups.” – Participant ID 2684 |
|  |  | Carer-led~discharge |  | 1 | “Once a patient no longer needs care as decided by their parent a discharge should be organised sooner.” – Participant ID 2653 |
|  |  | Discharge~process |  | 1 | “Discharge process” – Participant ID 760 |
|  |  | Discharge~timeline |  | 1 | “Now knowing when the doctor will call or when you have appointments. Not having a clear expectation for release, I spoke to many people who all had different assumptions on what criteria needed to be met to be released which was frustrating. Also, very invasive how if you didn't answer your cell phone, they called the room until you answered.” – Participant ID 523 |
|  |  | Home~COVID~test~before~discharge |  | 1 | “Home test before given me the clear to level home.” – Participant ID 1348 |
|  | **Information** | Education~of~service |  | 1 | “Original contact because I didn't know what RPAVirtual was, and was hesitant to get involved.” – Participant ID 2609 |
|  |  | Providing~call~summaries |  | 1 | “The doctors and nurses need to review the file notes of previous calls. Important to have good and accurate record management and ensure that a brief summary of call is captured so as not to get the patient to repeat the information each time. This is very important with regards to the list of medication being used by patients - keep up your great work and thank you so much to all your wonderful staff - you were of tremendous help and support to my elderly parents and myself - you guys are angels. A BIG THANK YOU to you all.” – Participant ID 2350 |
|  |  | Reduce~repeated~information |  | 1 | “The doctors and nurses need to review the file notes of previous calls. Important to have good and accurate record management and ensure that a brief summary of call is captured so as not to get the patient to repeat the information each time. This is very important with regards to the list of medication being used by patients - keep up your great work and thank you so much to all your wonderful staff - you were of tremendous help and support to my elderly parents and myself - you guys are angels. A BIG THANK YOU to you all.” – Participant ID 2350 |
|  | **Interpreter** | Ability~to~deny~interpreter |  | 1 | “My mum spoke Greek but also understood English. I'm her daughter who also is a carer for Mum and a registered nurse. Didn't require interpreter as previously stated she understood most, and o would interpret some she didn't understand.” – Participant ID 3338 |
|  |  | Asking~for~Interpreter |  | 1 | “People knowing this service is available. I wouldn't have known this service was available if I didn't click on a specific answer when I lodged my husband’s positive result on service now. Understanding what assistance is available may encourage more carers who require it to access it.” – Participant ID 2802 |
| **Reassurance** | **Family** | More~coordination~with~family |  | 2 | “At times it was hard with my elderly parents and not living with them ...to have the meetings.” – Participant ID 3582 |
|  |  | Emphasis~for~requiring~carer~presence |  | 1 | “If I wasn't there to look after my parents 24/7, they would not have been able to look after themselves. They would have needed a full-time nurse or be sent to hospital.” – Participant ID 2757 |
|  |  | Information~on~assistance~for~carers |  | 1 | “People knowing this service is available. I wouldn't have known this service was available if I didn't click on a specific answer when I lodged my husband’s positive result on service now. Understanding what assistance is available may encourage more carers who require it to access it.” – Participant ID 2802 |
|  |  | Updates~to~carer |  | 1 | “RPAVirtual also touched base with me (carer, not living at same address) and I was able to also relay information to mum and doctors.” – Participant ID 3334 |
| **Availability / accessibility** | **Personal** | Personalised~messages |  | 1 | “Advice from RPA Virtual addressed to Dear PATIENT. The close contact and then Casual Contact had same Mobile Number. There was NO WAY of knowing who the letter was meant for? Instructions referred to a Charger, but the device had a battery and did not need a charger?” – Participant ID 1562 |
|  | **Regular contact** | Asking~daily~care |  | 1 | “Asking daily care, very good” – Participant 469 |
|  |  | Consistent~follow-up |  | 1 | “Everything. The follow up has not been consistent. You call and they say they'll call back, and they don't. My family have been in home isolation for well over 14 days and I as the carer have had to email but no response. You need to sort this service out. I understand you're under pressure and maybe understaffed, but you need a proper process with follow up for isolated homes. This delay or lack of communication does not help those who need care in the household, but we cannot reach out to them because they're in isolation.” – Participant ID 1336 |
|  | **Scheduling** | Call~scheduling |  | 5 | “The care provided was ongoing over a period of a week. Care delivered was focused on my child, but I was also very unwell and because calls were unscheduled, they often woke me when I was finally able to rest/sleep (after caring for my child). It would be ideal to schedule calls and appointments if possible. Additionally, my child was very uncomfortable with being looked at and reviewed by a nurse/doctor over video and some nurses where not sympathetic and tried to continue checks via video calls despite causing my child to become upset and then hysterical. I felt these reactions should be better respected and approaches modified.” – Participant ID 3430 |
|  |  | Calling~two~times |  | 1 | “Daily two times check-up” – Participant 822 |
|  | **Availability** | Accessibility |  | 1 | “Accessibility and all of the above – Participant ID 3456 |
| **Clinical service** | **Clinical** | Faster~Covid~testing |  | 1 | Covid testing should be faster. The waiting time for a covid test order was long.” – Participant ID 960 |
|  |  | Hospital~in~the~home~visits |  | 1 | “They should send a nurse out to visit.” - Participant ID 3411 |
|  |  | Improved~decision-making |  | 1 | “That they should be able to make decisions if needed.” – Participant ID 3374 |
|  |  | Improved~record~management |  | 1 | “The doctors and nurses need to review the file notes of previous calls. Important to have good and accurate record management and ensure that a brief summary of call is captured so as not to get the patient to repeat the information each time. This is very important with regards to the list of medication being used by patients - keep up your great work and thank you so much to all your wonderful staff - you were of tremendous help and support to my elderly parents and myself - you guys are angels. A BIG THANK YOU to you all.” – Participant ID 2350 |
|  |  | Different~modalities~for~examination |  | 1 | “The care provided was ongoing over a period of a week. Care delivered was focused on my child, but I was also very unwell and because calls were unscheduled, they often woke me when I was finally able to rest/sleep (after caring for my child). It would be ideal to schedule calls and appointments if possible. Additionally, my child was very uncomfortable with being looked at and reviewed by a nurse/doctor over video and some nurses where not sympathetic and tried to continue checks via video calls despite causing my child to become upset and then hysterical. I felt these reactions should be better respected and approaches modified.” – Participant ID 3430 |
|  | **Escalation** | Improved~call~no-answer~protocol |  | 1 | “Now knowing when the doctor will call or when you have appointments. Not having a clear expectation for release, I spoke to many people who all had different assumptions on what criteria needed to be met to be released which was frustrating. Also, very invasive how if you didn't answer your cell phone, they called the room until you answered.” – Participant ID 523 |
|  |  | Improved~hospital~examination~escalations |  | 1 | “The initial oxygen reader I had was playing up however the staff were quick to organise another two to come out. This caused a quick trip to the hospital to confirm oxygen levels were ok (which they were). The trip to the hospital was if I'm honest a little inconvenient as my mother was not struggling to breathe and her colour and all other vitals were fine. I understand the doctor was doing her job and she was very professional and caring at the same time.” – Participant ID 3310 |
|  | **Pharmacy / medication** | rpavirtual~to~access~to~medication~lists |  | 2 | “The doctors and nurses need to review the file notes of previous calls. Important to have good and accurate record management and ensure that a brief summary of call is captured so as not to get the patient to repeat the information each time. This is very important with regards to the list of medication being used by patients - keep up your great work and thank you so much to all your wonderful staff - you were of tremendous help and support to my elderly parents and myself - you guys are angels. A BIG THANK YOU to you all.” – Participant ID 2350 |
|  |  | Improved~medication~management | Faster~medication~provision | 2 | “No follow up. Look at drugs patient is on and also understand patient may have other needs that need attending to when they have COVID.” – Participant ID 3112 |
|  | **Service** | More~resources |  | 1 | “It appears there is a lack of resources re. Follow up time, however it was excellent under the circumstances.” – Participant ID 2796 |
| **Staff** | **Staff** | Same~staff |  | 4 | “Having the same nurse for the entire period would have been optimal.” – Participant ID 341 |
| **Technology** | **Technology** | Improved~wearable~instructions |  | 2 | “Include on the note 'how to use Oximeter', that hands need to be warm to get accurate reading. Sometimes the zoom link wasn't received straight away due to technical glitch. Delay receiving oximeter, so RPA suggested hospital visit for patient which thankfully proved not needed. Otherwise, we were very grateful for RPAvirtual's support. Thank you!” – Participant ID 3101 |
|  |  | Contactless~wearables~delivery |  | 1 | “The delivery of the oximeter was not carried out by the transport provider - not did they attempt a contactless delivery. This was left to a staff member to do which while effective I felt was an improper use of the staff member's time.” – Participant ID 3258 |
|  |  | Improve~returning~equipment~process |  | 1 | “I have been trying to CONTACT you to return equipment.” – Participant ID 2978 |
|  |  | Improved~wearables~delivery |  | 2 | “The delivery of the oximeter was not carried out by the transport provider - not did they attempt a contactless delivery. This was left to a staff member to do which while effective I felt was an improper use of the staff member's time.” – Participant ID 3258 |
|  |  | Information~on~obtaining~wearables |  | 1 | “We didn't realise we were going to receive an oxygen monitor and had already bought one... So didn't take it” – Participant ID 2623 |
|  |  | Wearables~functionality | Working~wearables | 2 | “The health devices provided didn't work well were inconsistent Due to invalid reading an ambulance was sent to my home.” – Participant ID 1611 |
|  |  | Zoom link |  | 3 | “The zoom link. Would be great if I could just click and get straight to the meeting. Sometimes we get just a code.” – Participant ID 3395 |
|  |  | Preference~for~Zoom~app |  | 1 | “Zoom app was better as health one did not always.” work – Participant ID 244 |
| **Other** | **Everything** | Everything |  | 10 | “Everything” – Participant ID 1336 |
|  |  | Majority |  | 1 | “Majority of it” – Participant ID 1249 |

***Note:**

A single participant response can feature more than one key word or phrase.

Keywords related to Nothing (e.g., ‘nothing’, ‘great’, ‘helpful’, ‘good’) were excluded from the areas to improve word cloud as they did not mention any area to improve.

**Supplementary Table S4.** Supporting quotes of recommended actions based on carer suggestions for improvement or the reported best part of care

| **#** | **Recommended action based on carer suggestions for improvement or reported best part of care^1^** | **Supporting quotes from free-text responses** |
| --- | --- | --- |
| 1 | Use caring language and spend time with patients/carers answering questions to build trust, address concerns and ease uncertainty with carers. | - “Daily calls to monitor my young baby and providing valued and trusted advice to assist in his recovery” [Participant ID 2653] - “The unhurried consultations. The insightful questions asked by the team” [Participant ID 3013] - “Caring service” [Participant ID 2691] - “Peace of mind and reassurance.” [Participant ID 2757, 3110, 2819] - “It was reassuring that a health professional was monitoring mum and checking in everyday” [Participant ID 3213] - “Best possible care and can’t thank them enough. If they saw the patient is in distress they will take the time to put them at ease and comfort at the most difficult and embarrassing times... I can’t thank her enough for looking after my mum when she was struggling” [Participant ID 740] - “Patient had intellectual disability and limited verbal communication. The nurses were very adaptable as soon as they knew this and thoughtfully adjusted how they communicated with patient (via her advocate)” [Participant ID 2239] - “The care and understanding the needs of our clients, residents, workers. Thank you for your Amazing remarkable work to all.” [Participant ID 3158] - “When they check in on time and they give me the right words to encourage me.” [Participant ID1741] - “The fact that my mother received attention when we weren't expecting it. The took care of her needs and reassured her.” [Participant 2594] |
| 2 | Offer regular, personalised updates and practical advice/guidance to reassure carers about patient progress and care plans. | - “The ongoing support for my mother and the ability to ask questions. The whole process was professional, and all the clinical staff were patient and caring… A BIG thank you” [Participant ID 2623] - “Daily calls to monitor my young baby and providing valued and trusted advice to assist in his recovery” [Participant ID 2653] - “The staff I spoke to were very responsive, and went out of their way to answer all of my questions and provided me with information that I could then support my sister” [Participant ID 3110] - “Monitoring temperature. Checking observations 3 times a day over the phone.” [Participant ID 760] - “Having symptoms monitored daily by nurse and being informed if they were usual/expected. Having access to Dr when nurse or carer was especially concerned about progress of symptoms.” [Participant ID 2661] - “It was reassuring that a health professional was monitoring mum and checking in every day to see she was ok” [Participant ID 3213] |
| 3 | Identify patients who live alone and proactively engage carers (with patient consent) to reassure them that support is in place. | - “Knowing I wasn’t alone” [Participant ID 2680] - “The staff I spoke to were very responsive, and went out of their way to answer all of my questions and provided me with information that I could then support my sister” [Participant ID 3110] - “If I wasn't there to look after my parents 24/7 they would not have been able to look after themselves. They would have needed a full time nurse or be sent to hospital” [Participant ID 2757] |
| 4 | Clearly communicate the reasons behind care decisions, as carers value understanding the “why” to feel involved and reassured. | - “The follow up calls and ensuring recovery” [Participant ID 3338] - “Original contact because I didn't know what rpavirtual was and was hesitant to get involved” [Participant ID 2609] - “No one explained how or why my son was part of rpavirtual (from referral); the first call was totally out of the blue!” [Participant ID 3386] - “I wouldn't have known this service was available if I didn't click on a specific answer when I lodged my husbands positive result on service now. Understanding what assistance is available may encourage more carers who require it to access it.” [Participant ID 2802] - “The medical information. They knew what they were talking about. Very good medical knowledge.” [Participant ID 2089] - “More information on what could potentially be the cause of specific medical problems, not just one solution.” [Participant ID 2462] |
| 5 | Justify and communicate the use of interpreter services to prevent potential conflicts between patients and carers. | - “Perhaps to ask the patient if they need an interpreter” [Participant ID 2594] - “The 1st call nurse, she can speak my language Cantonese, and gave clearly information” [Participant ID 2427] |
| 6 | Streamline clear communications to reduce burden on carers, including reminders, pre-scheduled calls, and practical guides for virtual caregiving (e.g., at-home exercises, diet, pain management, and expected length of admission). | - “The patient was at home and easy for family to help” [Participant ID 3309] - “It would be ideal to schedule calls and appointments if possible.” [Participant ID 3430] - “We weren't always called when promised and I had to follow up. Often we were given conflicting information when speaking with different people.” [Participant ID 1133] - “If say you will call back in 20 mins call back then as we waited a lot for return calls.” [Participant ID 1997] - “The doctors and nurses need to review the file notes of previous calls. Important to have good and accurate record management and ensure that a brief summary of call is captured so as not to get the patient to repeat the information each time. This is very important with regards to the list of medication being used by patients - keep up your great work and thank you so much to all your wonderful staff - you were of tremendous help and support to my elderly parents and myself - you guys are angels.” [Participant ID 2350] - “Assurances and tips and guidance what to do - and also what red flags to look for” [Participant ID 2115] - “The information provided by rpavirtual was very helpful.” [Participant ID 2858] |
| 7 | Availability of daily 24-hour support with routine and after-hours contact options for timely assistance for patients and carers. | - “Daily calls to monitor my young baby and providing valued and trusted advice to assist in his recovery” [Participant ID 2653] - “Can't fault them. Whenever I called they were available to talk to. There was an incidence when I was in distress as my mum was struggling to breath. The Dr and nurse took the time to speak with me over the phone and kept talking to me until I was comfortable enough and not crying. They even comforted myself that my mum was progressing well. I highly rate them. The most difficult time of our lives and they are amazing people.” [Participant ID 740] - “Regular monitoring” [Participant 3146] - “Having symptoms monitored daily by nurse and being informed if they were usual/expected. Having access to Dr when nurse or carer was especially concerned about progress of symptoms.” [Participant ID 2661] |
| 8 | Coordinate scheduling with both patients and carers to maximise convenience and ensure carers are available to support. | - “The regularity of the contact. The unhurried consultations. The insightful questions asked by the team” [Participant ID 3013] - “Being involved although I live in Queensland” [Participant ID 3841] - “Care delivered was focused on my child, but I was also very unwell and because calls were unscheduled they often woke me.” [Participant ID 3430] - “If say you will call back in 20 mins call back then as we waited a lot for return calls.” [Participant ID 1997] - “At times it was hard with my elderly parents and not living with them ...to have the meetings” [Participant ID 3582] |
| 9 | Respond promptly to queries and callbacks to maintain carer trust and reduce stress during care delivery. | - “Communicating with the nurses and receiving call back from doctors” [Participant ID 1995] - “The staff I spoke to were very responsive, and went out of their way to answer all of my questions and provided me with information that I could then support my sister” [Participant ID 3110] - “Easy access. Helpful information.” [Participant ID 2536] - “It did take a few days to connect and they were my worse days but once we did the help and care was excellent” [Participant ID 1057] - “If say you will call back in 20 mins call back then as we waited a lot for return calls.” [Participant ID 1997] - “Time to respond to left messages for a call back” [Participant ID 2728; response to question area to improve] |
| 10 | Human interactions remain essential in virtual hospitals, value of healthcare workers as helpful, kind, and patient. | - “The doctors and nurses were very caring and provided lots of info” [Participant ID 3222] - “All the Doctors and Nurses were very nice and comforting and always stressed if any problems to call them ASAP” [Participant ID 1491] - “There was an incidence when I was in distress as my mum was struggling to breath. The Dr and nurse took the time to speak with me over the phone and kept talking to me until I was comfortable enough and not crying. They even comforted myself that my mum was progressing well. I highly rate them. The most difficult time of our lives and they are amazing people." [Participant ID 740] - “Nurses were really good and understanding” [Participant ID 2661] - “They were helpful and caring” [Participant ID 2189] - “Empathy was identified in the way we were supported through the process.” [Participant ID 2796] - “Excellent daily follow up. Wonderful service, not widely advertised/known but fantastic. Special thanks to a nurse who went out of her way to help, support my father to ensure his well-being. Please pass on my gratitude to her.” [Participant ID2839] |
| 11 | Consistency in healthcare workers allocation (e.g., same nurse and same doctors where able). | - “If possible to have the same nurses and doctor available at the time of consultation.” [Participant ID 3841] - “Having the same nurse for the entire period would have been optimal.” [Participant ID 341] |
| 12 | Conduct regular clinical check-ins and remote examinations, providing carers with confidence that patients are closely observed. | - “Great experience and was reason my elderly mother recovered much quicker” [Participant ID 3309] - “Felt very safe and confident we were doing everything to get a good result” [Participant ID 2767] - “Daily review very reassuring” [Participant 2641] - “Ability of doctor to visually diagnose patient and make decision to hospitalise patient” [Participant ID 2849] - “The close monitoring of my son and myself. Knowing that help was close by if needed.” [Participant ID 986] |
| 13 | Coordinate medications with patients, carers’, and pharmacy for safe medication management. | - “Medication & health device received within 24 hours” [Participant ID 2534] - “Organised to get me the antiviral medicine.” [Participant ID 2367] |
| 14 | Establish and communicate escalation pathways to help carers understand when and how to seek urgent care, preventing avoidable or delayed ED visits. | - “Monitoring symptoms and organised ambulance when needed” [Participant ID 2127] - “Felt very safe and confident we were doing everything to get a good result” [Participant ID 2767] - “The initial oxygen reader I had was playing up however the staff were quick to organise another two to come out. This caused a quick trip to the hospital to confirm oxygen levels were ok (which they were). The trip to the hospital was if I'm honest a little inconvenient as my mother was not struggling to breathe and her colour and all other vitals were fine. I understand the doctor was doing her job and she was very professional and caring at the same time.” [Participant ID 3310] - “They were helpful and were able to get flying squad out for a special need of a needed PCR” [Participant ID 3115] |
| 15 | Include carers in the referral and onboarding process to communicate expectations of technology checks, and care transitions (e.g., from GP or ED). | - “No one explained how or why my son was part of rpavirtual (from referral); the first call was totally out of the blue!” [Participant ID 3387] - “Include on the note 'how to use Oximeter', that hands need to be warm to get accurate reading. Sometimes the zoom link wasn't received straight away due to technical glitch. Delay receiving oximeter, so RPA suggested hospital visit for patient which thankfully proved not needed.” [Participant ID 3101] - “We didn't realise we were going to receive an oxygen monitor and had already bought one... So didn't take it...” [Participant ID 2623] |
| 16 | Include carers in discharge processes (e.g., coordinating wearable returns, connection to community GP). | - “Not having a clear expectation for release… which was frustrating.” [Participant ID 523] - “Once a patient no longer needs care as decided by their parent a discharge should be organised sooner” [Participant ID 2653] - “Not having a clear expectation for release, I spoke to many people who all had different assumptions on what criteria needed to be met to be released which was frustrating.” [Participant ID 523] - “Discharge process” [Participant ID 760; Response to question area to improve] |
| 17 | Checking technology functionality during onboarding and involve carers in setup (e.g., wearables functionality, Zoom/Microsoft teams link). | - “Knowledgeable management with technology to support” [Participant ID 3101] - “The health devices provided didn't work well were inconsistent. Due to invalid reading an ambulance was sent to my home” [Participant ID 1611] - “Include that hands need to be warm to get accurate reading (on oximeter). Sometimes the zoom link wasn't received straight away due to technical glitch.” [Participant ID 3101] - “Zoom link. Would be great if I could just click and get straight to the meeting” [Participant ID 3395] - “Zoom app was better as health one did not always work” [Participant 244] |
| 18 | Investment into reliable devices | - “The health devices provided didn't work well were inconsistent. Due to invalid reading an ambulance was sent to my home” [Participant ID 1611] - “The initial oxygen reader I had was playing up however the staff were quick to organise another two to come out. This caused a quick trip to the hospital to confirm oxygen levels were ok (which they were). The trip to the hospital was if I'm honest a little inconvenient as my mother was not struggling to breathe and her colour and all other vitals were fine. I understand the doctor was doing her job and she was very professional and caring at the same time.” [Participant ID 3310] - “Zoom” [Participant ID 1565; response to question best part of care] |
| 19 | Provide ongoing technical assistance (e.g., clear troubleshooting resources and ensure staff can offer basic IT support, considering varying levels of digital literacy). | - “Knowledgeable management with technology to support” [Participant ID 3101] - “Include that hands need to be warm to get accurate reading (on oximeter). Sometimes the zoom link wasn't received straight away due to technical glitch.” [Participant ID 3101] |

**Note:**

^1^Recommendations are listed in most frequently reported primary theme/service insight (i.e., Reassurance) to the least frequently reported primary theme/service insight (i.e., Usable Technology)
